# Gut microbiome–epigenetic crosstalk in obesity and type 2 diabetes: mechanisms, evidence, and translational opportunities

**DOI:** 10.3389/fmicb.2026.1805937

**Published:** 2026-03-31

**Authors:** Seham Saeed Alzahrani

**Affiliations:** Department of Biotechnology, College of Science, Taif University, Taif, Saudi Arabia

**Keywords:** DNA methylation, dysbiosis, epigenetics, gut microbiome, obesity, short-chain fatty acids, type 2 diabetes

## Abstract

Obesity and type 2 diabetes mellitus are closely linked metabolic disorders in which gut microbial alterations interact with host epigenetic regulation to influence inflammation, insulin sensitivity, and energy homeostasis. This review examines the gut microbiome–epigenetics interface in these conditions by integrating mechanistic evidence with a Scopus-based bibliometric analysis of publications from 2016 to 2025, thereby providing both disease-focused synthesis and field-level context. Bibliometric mapping identified 1,153 documents from 515 sources authored by 5,445 researchers, with a marked annual growth rate of 27.79%, indicating rapid expansion of this interdisciplinary area. Mechanistically, current evidence converges on three major epigenetic domains: DNA methylation, histone modifications, and non-coding RNA regulation. Microbiota-derived metabolites, particularly short-chain fatty acids, folate-related methyl donors, and other bioactive compounds, influence enzymes such as DNA methyltransferases and histone deacetylases, as well as downstream chromatin marks and microRNA networks relevant to metabolic dysfunction. In obesity and type 2 diabetes, recurrent findings include reduced abundance of butyrate-producing taxa and enrichment of pro-inflammatory or endotoxin-associated bacteria, although these patterns remain heterogeneous across populations, study designs, and analytical methods. Accordingly, the review emphasizes that phylum-level or taxa-level associations should be interpreted cautiously and that causality remains incompletely resolved. A key contribution of this review is the combined evaluation of mechanistic pathways, context-dependent microbial signatures, and translational limitations within a single framework. Overall, microbiome-targeted interventions remain promising but insufficiently validated, and progress toward clinical application will require longitudinal, multi-omics, and interventional studies that directly link specific taxa, metabolites, and epigenetic modifications.

## 1 Introduction

The escalating global prevalence of metabolic diseases represents one of the most pressing public health challenges of the 21st century (Garus-Pakowska, 2023). According to the International Diabetes Federation, 537 million adults were living with diabetes in 2021, with projections estimating this number will surge to 783 million by 2045, over 90% of whom have type 2 diabetes (Wu et al., 2023). Simultaneously, obesity has reached epidemic proportions, with more than 1.9 billion adults classified as overweight and over 650 million as obese globally, accounting for approximately 39% of the world’s population (Manisha, 2025). This convergence of obesity and type 2 diabetes underscores the need to define the molecular pathways that connect environmental exposures to metabolic dysfunction, rather than viewing these disorders only through the lens of caloric imbalance or genetic susceptibility.

Recent scientific advances have illuminated a tripartite relationship connecting gut microbiome, epigenetic regulation, and metabolic health (Kim et al., 2024). The human intestinal tract harbors trillions of microorganisms, whose collective activities contribute to nutrient metabolism, immune regulation, and intestinal barrier integrity (Sharma et al., 2019; Zhang Q. et al., 2025; Zhang Y. et al., 2025). Within this framework, the gut microbiome epigenetics axis has emerged as a mechanistically relevant model for explaining how dietary and environmental signals may become biologically embedded in host pathways that regulate inflammation, insulin sensitivity, adiposity, and energy homeostasis (Sharma et al., 2019; Kurhaluk et al., 2026). The seminal finding that the gut microbiota can exert profound effects on host physiology by modulating epigenetic mechanisms, including DNA methylation, histone post-translational modifications, and the regulation of non-coding RNA expression, has delineated a novel research axis in the field of metabolic diseases (Wu et al., 2022). Recent evidence demonstrates that diet-induced alterations in gut microbiome composition exert substantial epigenetic effects that modulate individual susceptibility to obesity and type 2 diabetes mellitus (Suleiman et al., 2025).

An expanding body of evidence indicates that microbial dysbiosis characterized by reduced bacterial alpha-diversity and disruptions in the relative abundance of commensal versus pathogenic taxa is strongly associated with both the onset and progression of metabolic dysfunction (Wei et al., 2021). Studies have revealed that individuals with type 2 diabetes and obesity exhibit distinctive gut microbial signatures, including decreased abundance of butyrate-producing bacteria such as *Faecalibacterium prausnitzii* and elevated levels of pro-inflammatory species (Sharma et al., 2019). These compositional shifts are associated with epigenetic modifications that ultimately influence gene expression patterns governing glucose metabolism, insulin sensitivity, and adiposity. At a mechanistic level, current evidence converges on three principal epigenetic domains through which microbial activity may influence metabolic disease: DNA methylation, histone modifications, and non-coding RNA regulation (Keating and El-Osta, 2015). Microbially derived metabolites, particularly the short-chain fatty acids butyrate, propionate, and acetate, function as endogenous inhibitors of histone deacetylases and as direct substrates or cofactors for chromatin-modifying enzymes, thereby modulating histone acetylation and other epigenetic marks (Zhang Q. et al., 2025; Zhang Y. et al., 2025). Additionally, gut bacteria influence DNA methylation patterns by modulating the availability of methyl donors through one-carbon metabolism, while also regulating the expression of microRNAs and long non-coding RNAs that control metabolic gene networks (Wu et al., 2023). Recent research further confirmed the bidirectional nature of this relationship, demonstrating that host genetic and epigenetic modifications reciprocally shape gut microbiome composition, creating a dynamic feedback loop that perpetuates metabolic health or disease (Ha et al., 2025). Furthermore, environmental factors including diet, air pollution, smoking, alcohol consumption, and antibiotic exposure significantly modulate this microbiome–epigenetic axis, providing both risk factors and therapeutic opportunities (Zhang Q. et al., 2025; Zhang Y. et al., 2025). The western diet, characterized by high saturated fat and simple carbohydrate content, has been shown to reduce gut microbial diversity and alter the Firmicutes-to-Bacteroidetes ratio, changes associated with pro-inflammatory epigenetic signatures that predispose individuals to obesity and insulin resistance (Sharma et al., 2019). Conversely, plant-based diets rich in dietary fiber promote beneficial microbial populations that produce metabolites with protective epigenetic effects (Bacaloni and Agrawal, 2025).

This intricate interplay between gut microbiota and epigenetic regulation represents a paradigm shift in our conceptualization of metabolic disease etiology. At the same time, the translational promise of this field should be interpreted with appropriate caution. Critically, the reversible and dynamic nature of epigenetic regulation distinguishes this pathway from genetic mutations, offering potential therapeutic opportunities for metabolic disease prevention and treatment through microbiome-targeted interventions, including prebiotics, probiotics, postbiotics, fecal microbiota transplantation, and precision dietary modifications (Nohesara et al., 2025). However, causality remains incompletely resolved, intervention responses are heterogeneous, and many studies do not yet directly integrate microbial, metabolic, and epigenetic endpoints in a sufficiently rigorous manner. Although several previous reviews have discussed microbiome epigenetic interactions in human disease, the present review is designed to make a more focused contribution by centering specifically on obesity and type 2 diabetes as interrelated metabolic disorders. Its conceptual novelty lies in integrating three levels of analysis within a single framework: first, the mechanistic effects of microbiota-derived metabolites on DNA methylation, histone modification, and non-coding RNA regulation; second, the links between dysbiosis, barrier dysfunction, inflammation, and insulin resistance in metabolic disease; and third, the translational implications of these findings for biomarkers, microbiome-directed interventions, and precision medicine. In addition, unlike a conventional narrative review alone, this manuscript incorporates a Scopus-based bibliometric overview of publications from 2016 to 2025 to situate the obesity-diabetes discussion within the broader evolution of microbiome epigenetics research and to identify the field’s shift toward mechanistic and clinically actionable themes.

## 2 Bibliometric overview

**Figure 1:**
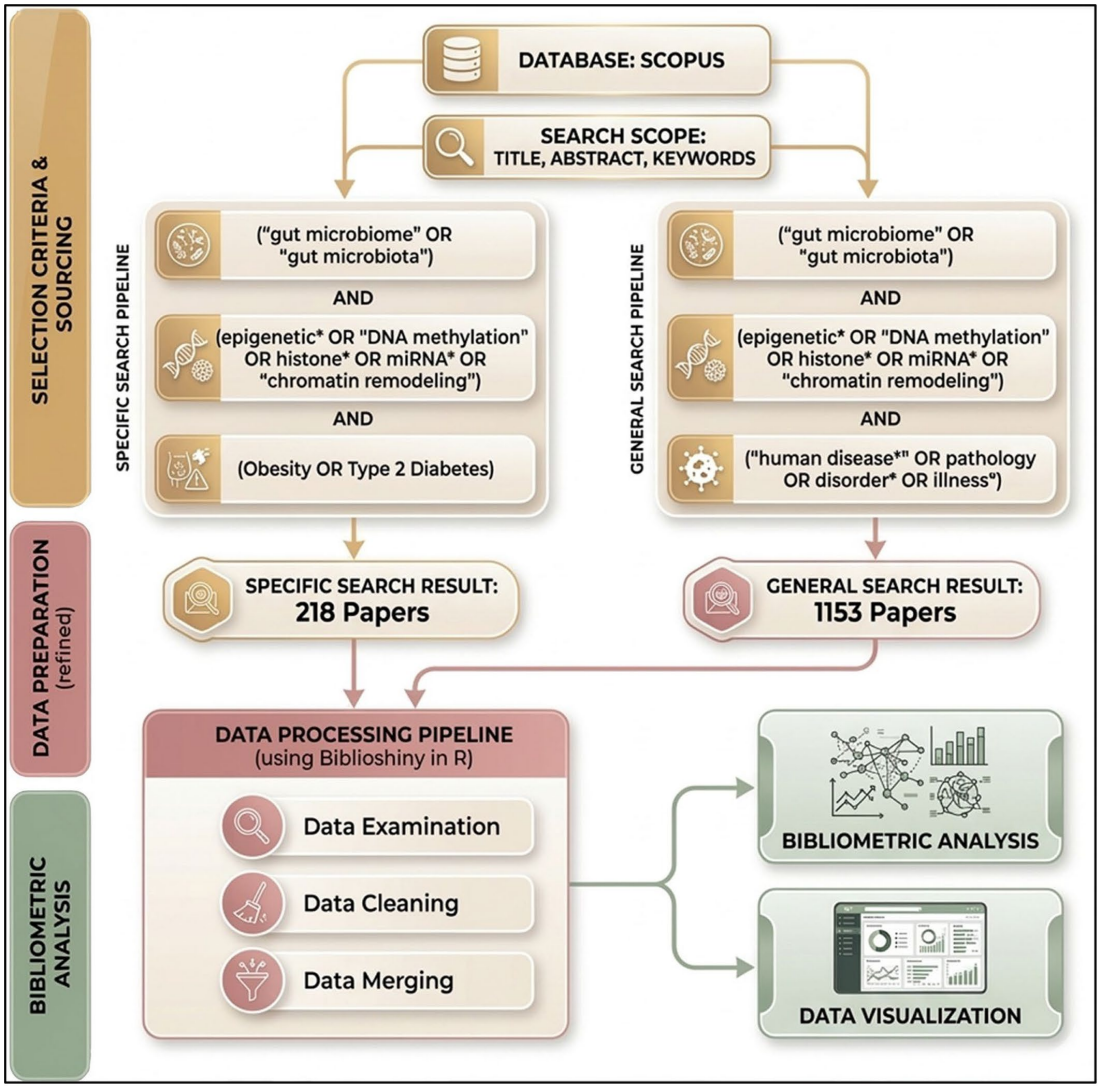
Schematic graph showing the process used for the bibliometric analysis.

A bibliometric analysis was conducted following the methodology described by Kinawy et al. (2024), as outlined in Figure 1. The analysis utilized publications indexed in Scopus and was based on the following general search query: (“gut microbiome” OR “gut microbiota”) AND (epigenetic* OR “DNA methylation” OR histone* OR miRNA* OR “chromatin remodeling”) AND (“human disease” OR pathology OR disorder OR illness*). This was subsequently refined using the specific term: AND (Obesity OR Type 2 Diabetes). The search results were exported as a RIS file and manually reviewed using Zotero to ensure all articles aligned with the thematic scope of the study. Further refinement and analysis were conducted using the Bibliometrix package in R-Studio, with Biblioshiny employed for the visualization of the results. The search was limited to documents published in English between 2016 and 2025. As detailed in Table 1, this strategy yielded a total of 1,153 publications, these documents appeared in 515 distinct sources, including peer-reviewed journals, books, and conference proceedings. Over the last decade, the annual growth rate of publications in this domain was 27.79%, indicating a rapid increase in scholarly interest at the interface of gut microbiome research and epigenetic mechanisms in human disease. The mean age of the documents was 3.74 years, and each document received an average of 51.53 citations, reflecting both substantial scientific impact and ongoing research activity in this field. In total, 5,445 authors contributed to the identified publications, whereas only 44 documents were single-authored, underscoring the highly collaborative nature of research in this area. The mean number of co-authors per document was 6.56, and 26.63% of the publications involved international collaboration, emphasizing the global scope of the research community investigating gut microbiome–epigenetic interactions in human pathology. With respect to document types, reviews (*n* = 687) and original research articles (*n* = 386) predominated, jointly accounting for more than 90% of the corpus. The dataset contained 9,892 keywords plus and 2,910 author-supplied keywords, underscoring the thematic breadth and the evolving conceptual landscape of research on the gut microbiome and epigenetic processes in human disease.

**Table 1:** Bibliometric characteristics of Scopus-indexed publications on the gut microbiome and epigenetic mechanisms in human diseases (2016–2025).

Description	Results
Main information about data	
Timespan	2016:2025
Sources (journals, books, etc.)	515
Documents	1,153
Annual growth rate %	27.79
Document average age	3.74
Average citations per doc	51.53
Document contents	
Keywords plus (ID)	9,892
Author’s keywords (DE)	2,910
Authors	
Authors	5,445
Authors of single-authored docs	44
Authors collaboration	
Single-authored docs	44
Co-authors per doc	6.56
International co-authorships %	26.63
Document types	
Article	386
Book	5
Book chapter	57
Conference paper	4
Editorial	5
Letter	1
Note	2
Review	687
Short survey	6

**Figure 2:**
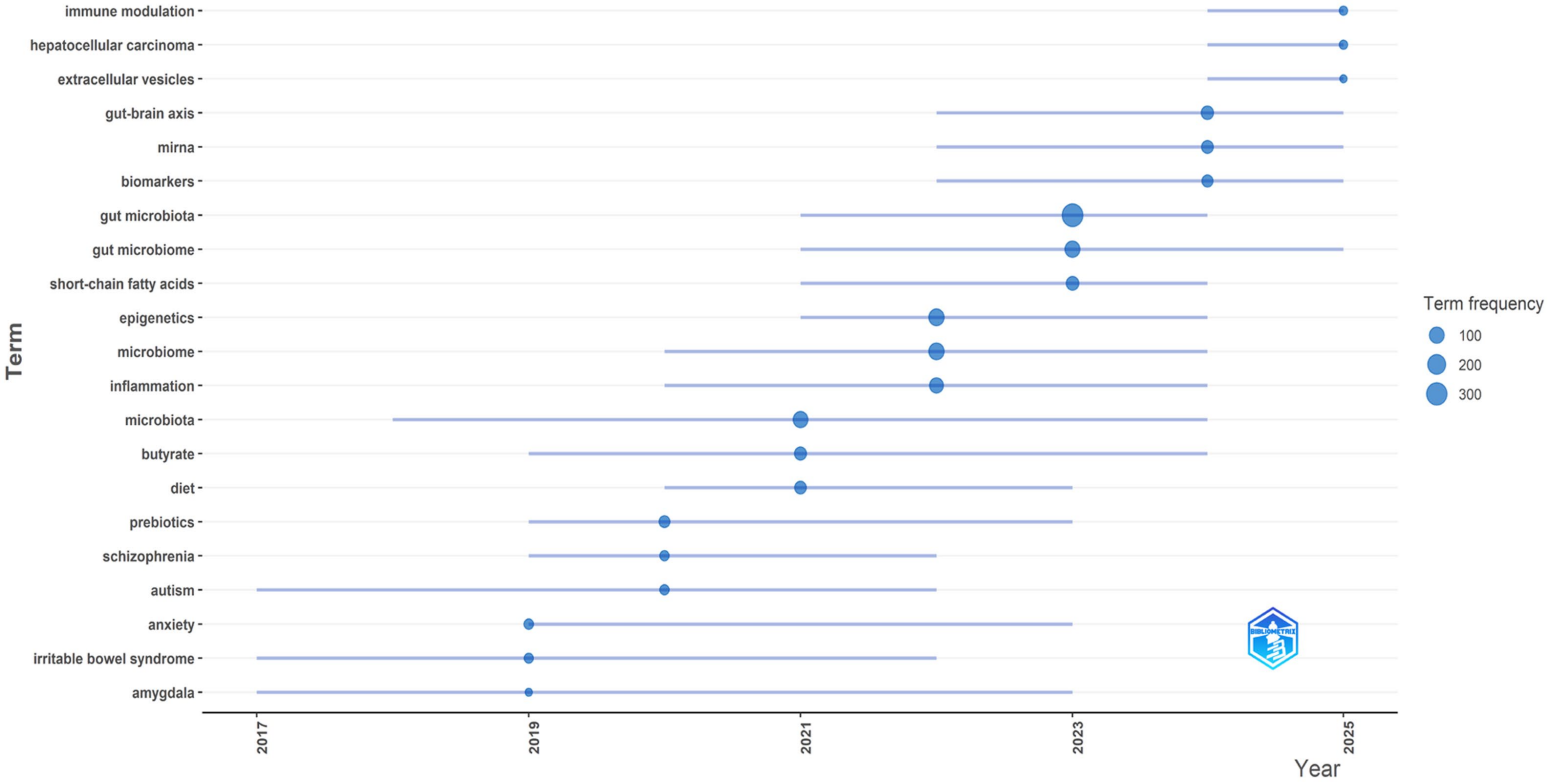
Temporal evolution of trending topics based on author keywords in Scopus-indexed publications on the gut microbiome/gut microbiota and epigenetic mechanisms in human diseases (2016–2025); bubble size represents keyword frequency, and connecting lines show the time span during which each term appears.

The trend-topics map (Figure 2) indicates that early-stage topics (2017–2019) are enriched for disease- or brain region-specific terms such as “amygdala,” “irritable bowel syndrome,” and “anxiety,” suggesting that initial investigations predominantly conceptualized microbiome–host interactions within narrowly defined clinical or behavioral contexts. In the 2019–2021 period, the emergence of “autism,” “schizophrenia,” and “prebiotics,” followed by diet- and metabolite-related terms (“diet,” “butyrate,” “microbiota”), reflects a shift toward modifiable environmental exposures and microbially derived products as putative mediators of host phenotypes. Between 2021 and 2023, more integrative and mechanistic umbrella terms (“inflammation,” “microbiome,” and “epigenetics”) become increasingly prominent, consistent with a consolidation of the field around immune-inflammatory signaling and epigenetic regulation as core explanatory frameworks. In the later period (2023–2025), “gut microbiome,” “gut microbiota,” and “short-chain fatty acids” exhibit high relative frequencies, while newer and emerging themes appear toward the end of the timeline, including “gut-brain axis,” “miRNA,” “biomarkers,” “extracellular vesicles,” “immune modulation,” and “hepatocellular carcinoma.” Taken together, the temporal trajectory suggests progression from heterogeneous, condition-specific applications to a more mechanistic and translational orientation, with a particular emphasis on inflammation, epigenetic modification, and microbially derived metabolites. The late emergence of “miRNA” and “extracellular vesicles” aligns with growing interest in inter-kingdom communication and molecular transport vehicles that may couple gut microbial activity to host gene-regulatory networks, while the increasing prominence of “biomarkers” points to a parallel movement toward clinically actionable molecular signature. Finally, the appearance of “immune modulation” and a cancer-specific term (“hepatocellular carcinoma”) near 2025 indicates both an expansion in disease scope and a more pronounced translational focus, wherein microbiome–epigenetic mechanisms are increasingly discussed within the domains of immunology and oncology.

**Figure 3:**
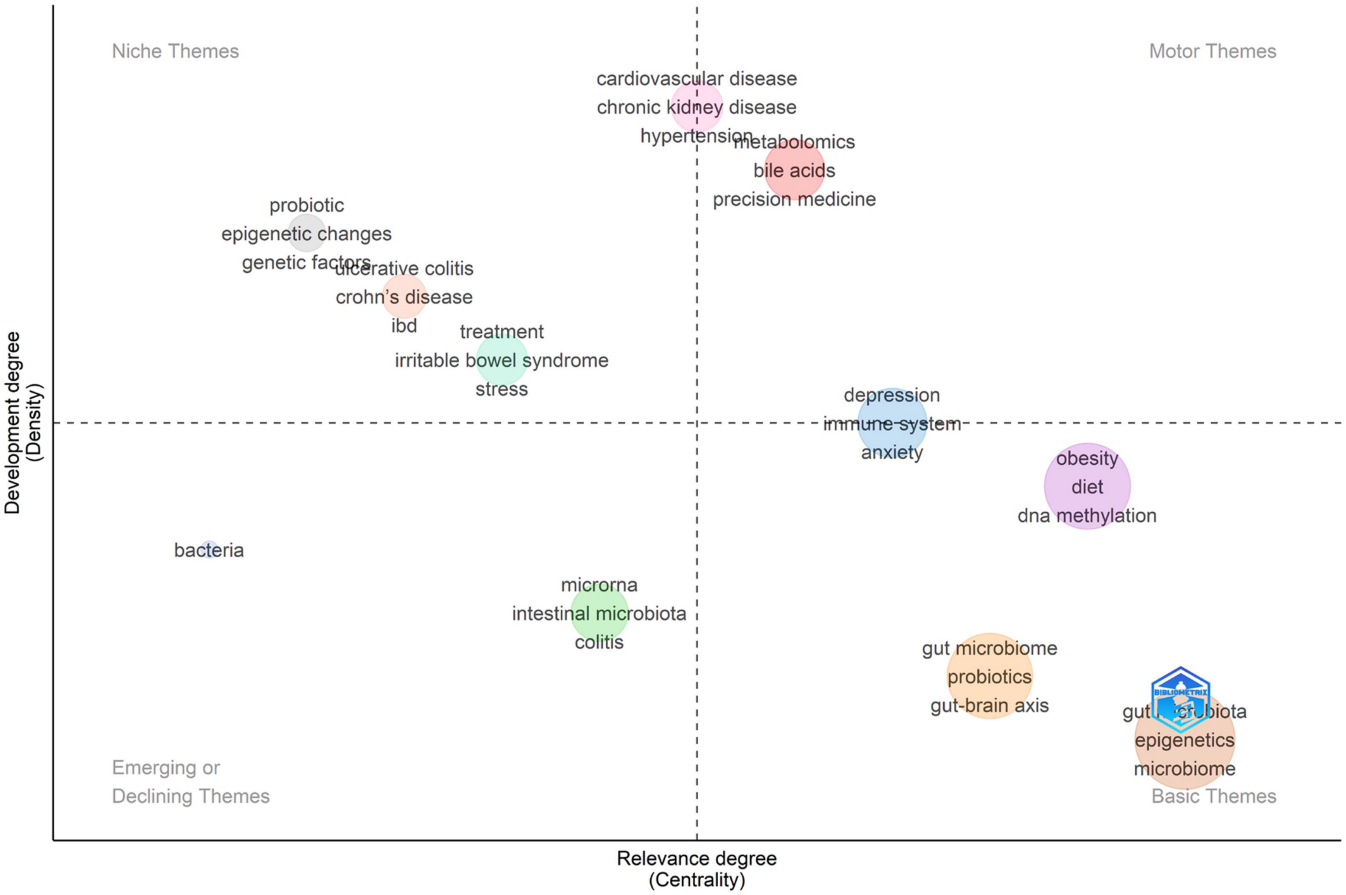
Thematic map (strategic diagram) of author-keyword co-occurrence clusters in gut microbiome–epigenetics research related to human diseases (2016–2025).

As illustrated in Figure 3, the largest and most central cluster is located in the basic-themes quadrant and is characterized by core field-defining terms such as “gut microbiota,” “microbiome,” and “epigenetics.” This configuration indicates that these constructs constitute the conceptual backbone of the dataset, while remaining relatively broad and internally heterogeneous rather than narrowly delimited or highly specialized. A second, still basic but more thematically focused cluster includes “gut microbiome,” “probiotics,” and “gut–brain axis,” pointing to a widely connected line of research that links modulation of the microbiome to neurobehavioral and neuroimmune outcomes, although its constituent subtopics are still dispersed across diverse methodological approaches and clinical conditions. In the motor-themes quadrant, clusters labeled with terms such as “metabolomics,” “bile acids,” and “precision medicine,” together with cardiometabolic and renal descriptors (e.g., “cardiovascular disease,” “chronic kidney disease,” “hypertension”), denote mature and highly interconnected themes that currently drive the development of the field. This positioning suggests that integrative multi-omics profiling and host–microbial metabolic axes (notably bile acid-mediated pathways) have emerged as central organizing frameworks in microbiome–epigenetic research with substantial translational potential for clinical practice. The niche-themes quadrant comprises clusters such as “probiotic,” “epigenetic changes,” and “genetic factors,” alongside gastrointestinal disease terms including “ulcerative colitis,” “Crohn’s disease,” and “IBD.” These clusters reflect well-developed yet comparatively specialized subfields that exhibit weaker integration into the broader thematic network. Their “isolated but mature” status indicates concentrated research communities that are deeply engaged within defined clinical domains particularly inflammatory bowel diseases while maintaining fewer cross-links to other disease areas represented in this dataset. Finally, themes in the lower-left quadrant (e.g., “bacteria,” as well as a cluster comprising “microRNA,” “intestinal microbiota,” and “colitis”) display both lower intercluster connectedness and lower internal development. This pattern is consistent with topics that are either emerging within this corpus or currently expanding less vigorously than other, more central areas of inquiry.

Beyond documenting the expansion of the publication record, these bibliometric patterns indicate a substantive reorientation of scientific priorities from predominantly descriptive microbiome profiling toward more mechanistic and translational lines of inquiry (more data are available in Supplementary material). The rising prominence of topics such as short-chain fatty acids, DNA methylation, microRNAs, biomarkers, and extracellular vesicles reflects increasing emphasis on identifying specific molecular mediators and clinically actionable signatures. At the same time, the uneven geographic distribution of research output highlights persistent limitations in global representation and, consequently, in the generalizability of current evidence. Collectively, these observations suggest that future progress will require not only continued mechanistic refinement, but also more diverse study populations, stronger international collaboration, and closer integration of multi-omics discovery with rigorous clinical validation. In this context, a clear understanding of the composition, ecological structure, and functional organization of the human microbiome is essential, because these features determine the metabolic capacities through which microbial communities may influence host epigenetic regulation and metabolic disease. Accordingly, the next section examines the human microbiome as a dynamic and functionally specialized system that underpins the mechanistic interactions discussed throughout this review.

## 3 Composition and functional organization of the human microbiome

**Figure 4:**
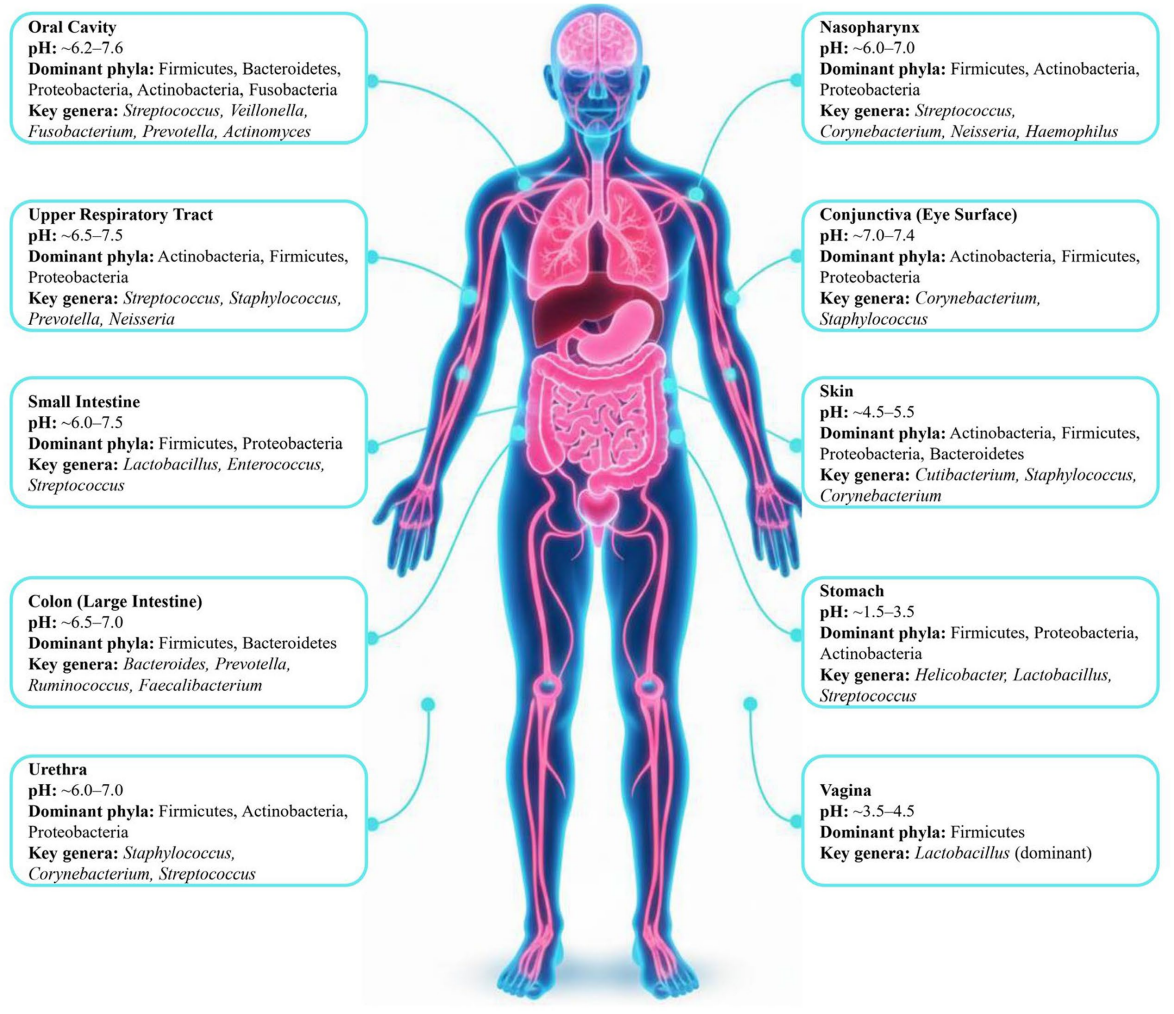
Spatial distribution of the human microbiota across principal anatomical sites, indicating site-specific pH ranges and the predominant bacterial phyla and genera.

The human microbiome constitutes a complex, highly dynamic, and densely interconnected ecological system composed of bacteria, archaea, fungi, viruses, and their collective genetic repertoire (Leonard and Toro, 2023). The term “microbiota” denotes the assemblage of microorganisms themselves, whereas “microbiome” encompasses these microorganisms together with their genomes and gene products, thereby capturing their aggregate functional potential within the host (O’Riordan et al., 2025). At any given time, the human body is estimated to harbor on the order of 500–1,000 bacterial species, and the collective gut microbiome alone contributes approximately 2–3 million genes, representing roughly 100–150-fold more genes than the 20,000 genes in the human genome (Zhu et al., 2010; Gilbert et al., 2018). Notably, microbiome composition is highly individualized and is shaped by an interplay of host genetic background, immune status, dietary patterns, lifestyle factors, and environmental exposures (Dabrowska and Witkiewicz, 2016). Microbial communities are heterogeneously distributed across the human body and demonstrate pronounced site specificity that is largely governed by local physicochemical parameters. Whereas Figure 4 depicts the relative abundances of the predominant microbial taxa across major anatomical niches, including the oral cavity, skin, stomach, vagina, and colon. At the phylum level, Firmicutes and Bacteroidetes constitute the dominant lineages across multiple body sites, whereas Actinobacteria, Proteobacteria, and Verrucomicrobia exhibit more restricted, niche-dependent distributions (Kennedy and Chang, 2020). While the gastrointestinal tract constitutes the most densely colonized microbial niche in the human body and harbors the majority of total microbial biomass (Hou et al., 2022). Within the colon, members of the phyla Firmicutes and Bacteroidetes predominate, reflecting their central roles in the fermentation of otherwise indigestible dietary components and in broader host-associated metabolic processes (Casciano, 2023). By contrast, the gastric environment, characterized by low pH and high acidity, supports a comparatively less diverse microbial community in which Firmicutes typically represent the dominant phylum (Rajilic-Stojanovic et al., 2020). Furthermore, the vaginal microbiome is defined by a pronounced dominance of Firmicutes, particularly species of the genus *Lactobacillus*, which are critical for maintaining an acidic vaginal pH and thereby inhibiting colonization by opportunistic and pathogenic microorganisms (Amabebe and Anumba, 2018). Cutaneous (skin-associated) microbial communities are enriched in Actinobacteria and Proteobacteria, a compositional pattern that reflects adaptation to heterogeneous microenvironments differing in moisture, oxygen availability, and sebum production (Bay et al., 2024). The oral cavity harbors a highly diverse and complex microbiota, consistent with its intricate anatomical architecture and continuous exposure to exogenous substrates and environmental inputs (Sedghi et al., 2021). At higher taxonomic resolution, each anatomical niche supports distinct microbial genera that are specifically adapted to its physicochemical and ecological conditions. The oral cavity is predominantly colonized by genera such as *Streptococcus*, *Abiotrophia*, *Peptostreptococcus*, and *Fusobacterium*, whereas the respiratory tract microbiota is characterized by the predominance of *Prevotella*, *Streptococcus*, *Staphylococcus*, *Neisseria*, and *Corynebacterium* (Schenck et al., 2016; Băncescu et al., 2020; Zhou et al., 2024). The gut microbiota is enriched in functionally critical taxa, including Bacteroides, Prevotella, and Ruminococcus, which are integral to host digestion, immune modulation, and metabolic homeostasis (Liu et al., 2019; Shah et al., 2025; Yang et al., 2025). Beyond bacteria, the gut ecosystem also encompasses fungi, bacteriophages, eukaryotic viruses, and methanogenic archaea, notably *Methanobrevibacter smithii* (Lozupone et al., 2012). Importantly, the relevance of microbiome composition to metabolic disease does not lie solely in the presence or absence of particular taxa, but in the functional capacities that those communities encode and express. Taxonomic configurations influence the availability of microbiota-derived metabolites, including short-chain fatty acids, methyl-donor-related compounds, and other low-molecular-weight signaling molecules, which may in turn affect host epigenetic regulation through modulation of DNA methylation, histone modifications, and non-coding RNA pathways (Turnbaugh et al., 2009; D’Aquila et al., 2020; Wu et al., 2021). Accordingly, variation in microbiome composition becomes mechanistically relevant when it alters metabolic pathway representation, substrate utilization, and metabolite output, rather than when it is interpreted only at a broad descriptive taxonomic level (Noecker et al., 2016). This distinction is particularly important in obesity and type 2 diabetes, where similar high-level taxonomic patterns may conceal substantial differences in functional potential and therefore in epigenetically relevant host exposure.

## 4 The human epigenetic link through microbial metabolites

The “human epigenetic bridge” can be conceptualized as a mechanistic interface through which the metabolic products of the gut microbiome couple environmental exposures, most prominently dietary inputs to host gene regulatory networks without changing the underlying DNA sequence, this coupling is mediated primarily via modulation of DNA methylation patterns, post-translational histone modifications, and non-coding RNA regulatory programs (Hullar and Fu, 2014; Wu et al., 2021). This conceptualization is consistent with models positing that the intestinal microbiome constitutes a “second genome,” whose metabolites interface with the host genome to induce stable epigenetic modifications that may shape long-term health trajectories (Romano and Rey, 2018). The bridge concept is consistent with the “human metagenome” perspective, wherein host biology reflects integrated human and microbial genetic and metabolic capabilities, rather than the host genome alone (Paul et al., 2015). A critical systems-level premise of this bridge is that the gut microbiome varies substantially in taxonomic composition across individuals while retaining comparatively stable functional and metabolic pathway representation at the community level (Human Microbiome Project Consortium, 2012). Twin-based and population-scale metagenomic analyses support the hypothesis that distinct microbial community compositions can nevertheless converge on a shared functional core at the gene and pathway levels. Furthermore, deviations from this conserved functional repertoire are associated with specific physiological phenotypes, including obesity (Turnbaugh et al., 2009). While healthy individuals exhibit wide taxonomic variation alongside more stable metabolic pathway carriage (Human Microbiome Project Consortium, 2012). This functional stability provides a plausible ecological basis for metabolite-mediated epigenetic effects: if metabolic capacities are conserved, then metabolite exposures capable of shaping epigenetic regulation may be recurrent across individuals even when microbial taxa differ (Human Microbiome Project Consortium, 2012).

### 4.1 Mechanistic pathways of microbiome–epigenetic regulation

The epigenetic bridge operates through three principal mechanistic pathways: (i) direct modulation of chromatin-modifying enzyme activity by microbial metabolites, (ii) alteration of substrate and cofactor availability for epigenetic reactions, and (iii) regulation of non-coding RNA expression and function. These pathways converge on DNA methylation patterns, histone post-translational modifications, and microRNA-mediated gene silencing, collectively shaping transcriptional programs relevant to metabolic homeostasis. Short-chain fatty acids (SCFAs) principally acetate, propionate, and butyrate are key microbial metabolites generated through the fermentation of dietary fiber and resistant starch. Butyrate acts as a potent inhibitor of zinc-dependent class I and II histone deacetylases (HDACs) by occupying the catalytic pocket coordinated by zinc ions, thereby blocking substrate access and preventing deacetylation (van der Hee and Wells, 2021). This HDAC inhibition enhances histone acetylation at lysine residues, leading to a more relaxed chromatin conformation and promoting transcription factor binding at genomic loci implicated in insulin sensitivity, expression of anti-inflammatory cytokines (e.g., IL-10, TGF-β), and synthesis of barrier-stabilizing tight junction proteins such as claudin and occludin (Davis-Richardson and Triplett, 2015). Moreover, butyrate downregulates the expression of DNA methyltransferases (DNMTs) specifically DNMT1, DNMT3a, and DNMT3b in obesity models, thereby attenuating hypermethylation at promoters of critical metabolic genes, including adiponectin and resistin (Rukavina Mikusic et al., 2025). Propionate contributes to epigenetic remodeling of DNA methylation patterns by modulating intracellular S-adenosyl-L-methionine (SAM) availability and/or by signaling through the G-protein-coupled receptors GPR41 and GPR43, which initiate downstream signaling cascades that alter chromatin architecture via transcription factor activation (Liu, 2025). Acetate primarily exerts indirect effects by entering the hepatic acetyl-CoA pool, thereby increasing substrate availability for histone acetyltransferases (HATs) and promoting lysine acetylation at regulatory regions of genes involved in lipid oxidation, such as FGF21 (Miro-Blanch and Yanes, 2019; Zhang et al., 2025; Zhang et al., 2025). Beyond their canonical role as histone deacetylase inhibitors, short-chain fatty acids also promote a spectrum of non-acetyl histone acylations that constitute an emerging layer of epigenetic regulation. Metabolites such as crotonate and propionate are converted into their corresponding acyl-CoA derivatives (crotonyl-CoA and propionyl-CoA), which function as alternative acyl-donor substrates for histone-modifying enzymes. Multiple histone acetyltransferases (HATs), including CBP/p300, exhibit substrate promiscuity toward diverse acyl-CoA species and thereby catalyze the deposition of non-acetyl acyl marks. These non-canonical acylations are selectively recognized by specialized reader proteins that are distinct from classical acetyl-lysine-binding bromodomains (Miro-Blanch and Yanes, 2019). The resulting chromatin modifications reprogram transcriptional networks, including those governing lipid oxidative metabolism, and may contribute to population-specific phenotypic variability whereby equivalent elevations in bulk SCFA levels do not yield uniform metabolic benefits. SCFA concentration is a critical determinant of their mechanistic and translational effects. *In vitro* studies demonstrate that millimolar SCFA concentrations comparable to those found in the colonic lumen act predominantly via direct HDAC inhibition to induce expression of lipogenic genes, such as fatty acid synthase (FASN), with the potential to promote adipogenesis. In contrast, lower micromolar concentrations characteristic of systemic circulation primarily signal through G protein-coupled receptor (GPCR) pathways, enhancing secretion of glucagon-like peptide-1 (GLP-1) and peptide YY (PYY), suppressing appetite, and improving insulin sensitivity without concomitant pro-lipogenic activity (Liu, 2025). This concentration-dependent dichotomy presents a major challenge for extrapolating cell culture data to *in vivo* contexts and for designing dietary or probiotic interventions that seek to harness SCFA-mediated metabolic benefits.

Active DNA demethylation pathways mediated by ten-eleven translocation (TET) dioxygenases represent a critical yet comparatively undercharacterized dimension of microbiome–epigenome crosstalk. TET enzymes catalyze the iterative oxidation of 5-methylcytosine (5mC) to 5-hydroxymethylcytosine (5hmC), thereby initiating replication-independent reversal of cytosine methylation states. Within the context of metabolic inflammation, lipopolysaccharide (LPS)-induced oxidative stress modulates TET catalytic activity through its dependence on α-ketoglutarate as a co-substrate, leading to locus-specific enrichment of 5hmC at promoters and enhancers of pro-inflammatory cytokine genes. This epigenetic remodeling underlies the sustained transcriptional poising characteristic of “trained immunity” or “inflammatory memory” (Nelson and Friedman, 2024; Rukavina Mikusic et al., 2025). In macrophage culture systems exposed to repeated LPS stimulation, investigators have documented a persistent depletion of the repressive histone mark H3K27me3 at pro-inflammatory cytokine promoters, accompanied by increased histone acetylation and TET-dependent DNA demethylation. Notably, these chromatin and DNA methylation changes are maintained even following withdrawal of the inflammatory stimulus (Rukavina Mikusic et al., 2025), indicating durable epigenetic reprogramming. Despite their mechanistic and therapeutic relevance particularly for the development of reversible epigenetic interventions in metabolic disease these active demethylation processes remain substantially less elucidated than *de novo* and maintenance DNA methylation pathways.

A nuanced understanding of microbiome–epigenetic interactions requires acknowledgment that phylum-level taxonomic indicators most notably the Firmicutes/Bacteroidetes ratio are inadequate predictors of epigenetic outcomes in the absence of functional characterization. Metabolic output at the strain level exhibits considerable heterogeneity within individual phyla; for example, not all expansions of Firmicutes are associated with improved SCFA profiles. Specific clostridial taxa produces butyrate species with potent histone deacetylase inhibitory activity, whereas others predominantly generate propionate, which differentially modulates lipid metabolism (Yu et al., 2025). Analogously, within the phylum Bacteroidetes, bile salt hydrolase (BSH) activity is not uniform: BSH enzymes encoded by Firmicutes and Actinobacteria catalyze deconjugation of a broad spectrum of bile salts, while Bacteroidetes-derived BSH activity is largely confined to tauro-conjugated substrates (Alkhammash et al., 2022). These functional distinctions imply that ostensibly similar phylum-level community structures can possess markedly divergent capacities for methyl-donor biosynthesis, HDAC inhibition, and host receptor engagement, thereby generating distinct epigenetic signatures.

Bile acids (BAs) function as signaling molecules that extend beyond their canonical role in facilitating lipid digestion. Microbial biotransformation of primary BAs into secondary derivatives deoxycholic acid (DCA), lithocholic acid (LCA), and hyocholic acid (HCA) modifies the ligand repertoire available for engagement of nuclear and membrane receptors. Activation of the farnesoid X receptor (FXR) typically attenuates BA synthesis via small heterodimer partner (SHP)-dependent repression of cholesterol 7α-hydroxylase (CYP7A1) and concurrently modulates glucose homeostasis through transcriptional regulation of gluconeogenic genes. In parallel, stimulation of the Takeda G-protein-coupled receptor 5 (TGR5) elevates intracellular cAMP levels, initiating protein kinase A (PKA)-dependent phosphorylation cascades that recruit CREB and histone acetyltransferases (HATs), thereby enhancing histone acetylation marks such as H3K27ac at loci encoding metabolic regulators (Liu, 2025). The coexistence of multiple BA species *in vivo* renders receptor-binding dynamics highly competitive, complicating mechanistic predictions; competition for FXR and TGR5 can attenuate the chromatin-level impact of individual BA species. Chenodeoxycholic acid (CDCA) is a potent FXR agonist; its increased abundance following supplementation has been associated with enhanced mitochondrial uncoupling in brown adipocytes but simultaneously suppress histone acetylation at genomic regions controlling lipid biosynthetic pathways. In contrast, HCA exerts dual activity by activating TGR5 while antagonizing FXR, thereby augmenting glucagon-like peptide-1 (GLP-1) secretion and potentially sustaining histone acetylation at genes that promote glucose utilization (Liu, 2025). Moreover, tissue-specific receptor expression profiles introduce an additional layer of complexity in translating BA signaling into chromatin and metabolic outcomes. Hepatic chromatin responses to BA-mediated FXR/TGR5 signaling differ markedly from those in adipose tissue, reflecting distinct receptor abundance and signaling context across these compartments (Alkhammash et al., 2022).

Histone acetyltransferases (HATs), particularly members of the p300/CBP family, catalyze the transfer of acetyl groups from acetyl-CoA to *ε*-amino groups of lysine residues on histone tails, thereby promoting chromatin relaxation and facilitating transcriptional activation. Microbial metabolites modulate HAT recruitment and activity through multiple, partially interdependent mechanisms. Acetate produced by bacterial fermentation contributes to hepatic acetyl-CoA pools, thereby increasing substrate availability for p300/CBP-mediated histone acetylation at loci such as FGF21, a hormone that governs lipid oxidation and glucose homeostasis (Zhang Q. et al., 2025; Zhang Y. et al., 2025). In addition, activation of GPR41/43 by SCFAs initiates intracellular signaling cascades that enhance p300/CBP recruitment to genes involved in energy expenditure via CREB phosphorylation-dependent pathways (Li et al., 2014). In diet-induced obesity models characterized by enrichment of butyrate-producing genera (e.g., Roseburia, Faecalibacterium), hepatic chromatin immunoprecipitation analyses reveal increased histone acetylation at the promoters of PPARγ coactivator-1α (PGC-1α) and GLUT4, accompanied by augmented p300 occupancy; these epigenetic alterations correlate with enhanced mitochondrial oxidative capacity and improved insulin-stimulated glucose uptake (Woo and Alenghat, 2022). *In vitro* studies further support a causal relationship: hepatocytes exposed to physiologically relevant acetate concentrations exhibit rapid enrichment of H3K27ac at genes that suppress gluconeogenesis, coincident with p300/CBP recruitment, whereas pharmacological inhibition of HAT activity abrogates acetate-induced transcriptional upregulation despite unchanged acetyl-CoA availability (Zhang Q. et al., 2025; Zhang Y. et al., 2025).

Histone deacetylases (HDACs) constitute a heterogeneous enzyme family with isoform-specific functions that are critically involved in the regulation of metabolic homeostasis. HDAC3, a class I HDAC, exerts a pivotal role in hepatic lipid metabolism. Genetic ablation of HDAC3 in mice with conventional diet-induced obesity attenuates body weight gain, lowers circulating triglyceride concentrations, and enhances insulin sensitivity; however, the same deletion concomitantly increases vulnerability to intestinal injury under conditions in which microbiota-derived signals that modulate lipid metabolism are perturbed (Ha et al., 2025). HDAC6, a class IIb HDAC, is implicated in the regulation of leptin signaling and inflammatory pathways. Pharmacological inhibition of HDAC6 in murine models of diet-induced obesity promotes weight reduction through restoration or augmentation of leptin signaling, while simultaneously improving glucose tolerance and suppressing NLRP3 inflammasome-dependent pyroptosis, thereby conferring additional protective effects in the context of diabetic nephropathy (Kiełbowski et al., 2025). Collectively, these isoform-specific actions emphasize that therapeutic approaches targeting HDACs must be highly selective to minimize off-target effects while maintaining or enhancing their beneficial metabolic outcomes.

DNA methyltransferases display isoform-specific functional specializations: DNMT1 predominantly preserves pre-existing methylation patterns during DNA replication, whereas DNMT3A and DNMT3B catalyze *de novo* methylation at previously unmethylated genomic loci (Suárez et al., 2023). Distinct microbial metabolites exert differential regulatory effects on these isoforms. Butyrate exposure diminishes nuclear DNMT1 abundance in intestinal epithelial cell cultures and concomitantly induces selective hypomethylation of CpG sites within promoters of genes involved in epithelial barrier function. In contrast, folate supplementation partially restores global 5mC levels but does not fully reinstate site-specific transcriptional repression when the availability of one-carbon cycle cofactors remains incongruent with local chromatin accessibility constraints (Shock et al., 2021). Moreover, lipopolysaccharide (LPS) derived from Gram-negative bacterial overgrowth downregulates DNMT3A transcription via Toll-like receptor 4 (TLR4) dependent signaling pathways and modifies cofactor requirements within methionine cycle enzymes. In animal models, these perturbations are associated with hypomethylation and consequent overexpression of key gluconeogenic enzymes, including phosphoenolpyruvate carboxykinase (PEPCK) and glucose-6-phosphatase (G6Pase), thereby contributing to fasting hyperglycemia (Devaux and Raoult, 2018).

**Figure 5:**
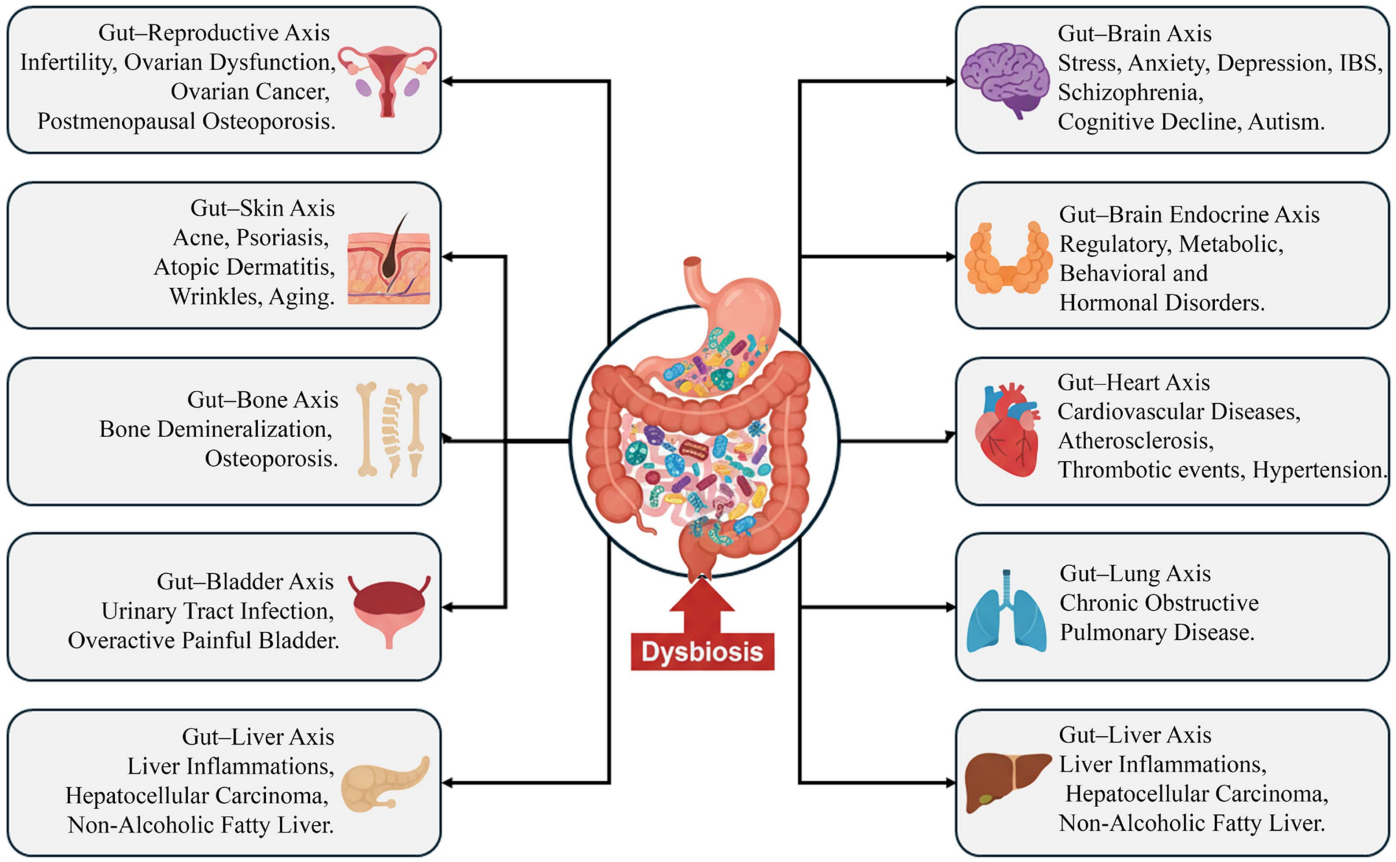
Conceptual model of how microbial dysbiosis perturbs epigenetic regulatory pathways across interconnected gut-organ axes and contribute to systemic disease processes.

MicroRNAs (miRNAs) represent a pivotal interface within microbiome–epigenetic crosstalk, functioning both as effectors that respond to microbial metabolites and as regulators that modulate bacterial gene expression. SCFAs regulate miRNA expression through several molecular mechanisms. In particular, butyrate alters the methylation status of miRNA promoter regions via HDAC inhibition, thereby enhancing the transcription of miRNAs implicated in anti-inflammatory processes and lipid metabolism (Bishop et al., 2017). Dysbiotic conditions characterized by reduced abundance of SCFA-producing taxa are associated with diminished expression of protective miRNAs (e.g., miR-126) and concomitant upregulation of pro-inflammatory miRNAs (e.g., miR-155). These miRNAs target key regulators of vascular homeostasis and insulin sensitivity, linking microbiota composition to cardiometabolic risk (Drago et al., 2025). Importantly, this regulatory network is bidirectional a concept that substantially reframes current models of host–microbe interactions. Host-derived miRNAs can be taken up by bacterial cells and directly modulate bacterial gene transcription. For example, miR-515-5p has been shown to influence the growth dynamics of *Escherichia coli* and *Fusobacterium nucleatum* by binding to and regulating specific bacterial transcripts. Additional host miRNAs modulate bacterial gene networks involved in quorum sensing, biofilm formation, and central metabolic pathways (Zhang Q. et al., 2025; Zhang Y. et al., 2025). This reciprocal exchange establishes a dynamic feedback loop in which the host epigenetic landscape shapes microbial community structure and function, while microbially derived metabolites, in turn, influence host miRNA expression profiles. Evidence from gnotobiotic models supports this concept. Germ-free mice display distinct intestinal epithelial miRNA signatures relative to specific pathogen-free counterparts; subsequent colonization alters the expression of multiple miRNAs, including the upregulation of miR-21-5p targeting barrier function-related genes such as ARF4 (Nai et al., 2025). Similarly, in rodent models fed a high-fat diet, the liver exhibits overexpression of miR-103 and miR-107, which is associated with impaired insulin sensitivity through suppression of caveolin-1. Microbiota alterations further stabilize or exacerbate these miRNA changes via inflammatory mediators that impact transcriptional regulation (Li et al., 2020). Figure 5 synthesizes these interconnected pathways, illustrating how microbial dysbiosis can perturb epigenetic regulatory networks across multiple gut-organ axes, thereby contributing to systemic disease processes. This conceptual model underscores that the mechanisms detailed above HDAC inhibition, TET-dependent DNA demethylation, miRNA regulation, and bile acid signaling do not operate in isolation but rather constitute an integrated network through which the microbiome shapes host physiology.

### 4.2 Conceptual framework of the microbiome–epigenome interface

Extensive microbiome profiling demonstrates that microbial community composition is niche-specialized and exhibits substantial inter-individual variability even among clinically healthy subjects. Nevertheless, metagenomically inferred metabolic pathways remain comparatively stable across individuals, suggesting that the potential for metabolite production is preserved despite pronounced taxonomic heterogeneity (Human Microbiome Project Consortium, 2012). Consistent with this, analyses of obese versus lean twins indicate that obesity associates with phylum-level shifts, reduced diversity, and altered representation of microbial genes and metabolic pathways (Turnbaugh et al., 2009). Recent integrative reviews of global microbiome research further underscore that high-throughput sequencing technologies have unveiled extensive microbial diversity across human populations and physiological or pathological states. Collectively, these findings reinforce the overarching conceptual framework that human metabolic phenotypes constitute a composite of host-derived and microbiota-derived functional attributes (Gupta et al., 2017). Whereas, host genetic variation exerts a quantifiable influence on gut microbiome composition, including the heritable abundance of specific microbial taxa. This observation suggests that genetically mediated alterations in microbial community ecology can give rise to corresponding differences in microbiome-derived metabolite production, which in turn may elicit distinct downstream epigenetic modifications (Cuevas-Sierra et al., 2019). The interaction between genetic background, diet, and gut microbiota has been repeatedly highlighted in the context of obesity and metabolic syndrome risk, providing a plausible route through which inherited factors indirectly influence epigenetic states by modulating microbiome-derived metabolite exposures (Cuevas-Sierra et al., 2019).

## 5 Obesity and metabolic disorders, including diabetes mellitus

Obesity has been associated with altered gut microbiota composition, reduced bacterial diversity, and altered representation of microbial genes and metabolic pathways, establishing a microbiome-level substrate for altered metabolite exposure relevant to host epigenetic regulation (Turnbaugh et al., 2009; Harakeh et al., 2016). Reviews emphasize that gut microbiome alterations can be involved in metabolic disease pathogenesis via induction of epigenetic changes (DNA methylation, histone modifications, noncoding RNAs), with microbiota-produced metabolites (e.g., SCFAs, folates, biotin, TMAO) participating in these regulatory processes (Cuevas-Sierra et al., 2019; Sharma et al., 2019). Empirical studies link gut microbiota composition to global DNA methylation patterns in obesity and highlight microbiota-produced folic acid and polyamines as plausible contributors via carbon metabolism and methylation pathways (Ramos-Molina et al., 2019; Salas-Perez et al., 2023). While, recent work examining crosstalk between gut microbiota and epigenetic markers in obesity underscores that both microbiota composition and epigenome are strongly impacted by lifestyle especially dietary patterns reiterating that microbiome changes can may influence epigenetic changes involving DNA methylation, non-coding RNAs, and histone modifications (Salas-Perez et al., 2023). These convergent findings support an integrated model where obesity risk reflects interactions among diet, microbiome ecology, microbial metabolite profiles, and epigenetic regulation potentially modulated by host genetic background (Goodrich et al., 2014; Cuevas-Sierra et al., 2019).

### 5.1 Microbiome-mediated epigenetic mechanisms in immunometabolic regulation

**Figure 6:**
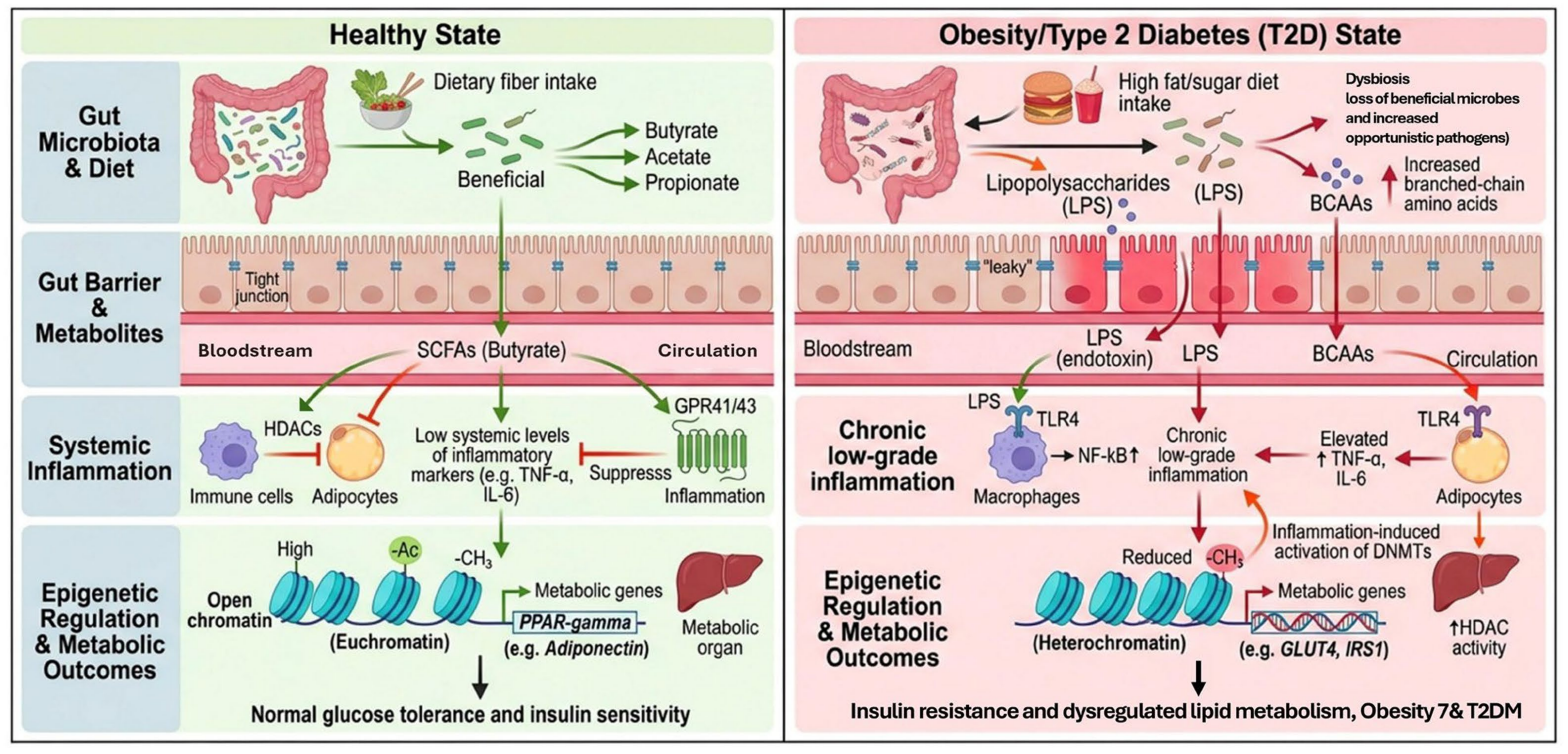
Microbiome–epigenetic mechanisms in health vs. obesity/T2D. Healthy state: dietary fiber promotes SCFA production, HDAC inhibition, and GPR41/43 signaling, suppressing inflammation and maintaining insulin sensitivity. Obesity/T2D: dysbiosis increases LPS and BCAAs, causing “leaky gut,” TLR4-NF-κB activation, chronic inflammation, and DNMT/HDAC-driven epigenetic repression of metabolic genes (GLUT4, IRS1).

Beyond compositional alterations in the microbiota, accumulating evidence implicates isoform-specific dysregulation of epigenetic enzymes in the pathogenesis of obesity and type 2 diabetes mellitus. Histone deacetylase 3 (HDAC3), which is abundantly expressed in metabolically active tissues, functions as an integrative node for circadian and nutritional cues to orchestrate hepatic lipid homeostasis. In diet-induced obesity, HDAC3 activity is elevated at the promoters of lipogenic genes and concomitantly reduced at loci governing fatty acid β-oxidation, thereby promoting hepatic steatosis (Ha et al., 2025). In contrast, inhibition of HDAC6 exerts metabolically beneficial effects via distinct, tissue-specific mechanisms, including potentiation of leptin receptor signaling in the hypothalamus, enhancement of GLUT4 translocation in adipose tissue, and attenuation of NLRP3 inflammasome-dependent interleukin-1β production in macrophages (Kiełbowski et al., 2025). Collectively, these isoform-specific activities indicate that targeted modulation of individual HDACs rather than non-selective, pan-HDAC inhibition may provide therapeutic benefit while reducing oncogenic risk associated with broad-spectrum epigenetic perturbation. The contrasting epigenetic outcomes between healthy and diseased states are illustrated schematically in Figure 6.

Chronic, low-grade inflammation that is characteristic of obesity appears to involve epigenetic priming of immune cells mediated by TET (ten-eleven translocation) enzyme dependent DNA demethylation. Adipose tissue macrophages from individuals with obesity exhibit sustained enrichment of 5hmC at the promoters of TNF-α and IL-6, thereby preserving a transcriptionally poised state even following partial weight loss or metabolic improvement (Rukavina Mikusic et al., 2025). This “trained immunity” phenotype is proposed to arise from prior exposure to LPS during episodes of gut dysbiosis and contribute to the difficulty of fully reversing established insulin resistance. At the mechanistic level, LPS-induced activation of NF-κB recruits TET enzymes to defined genomic loci, where α-ketoglutarate-dependent oxidation facilitates maintenance of an open chromatin configuration at pro-inflammatory genes (Nelson and Friedman, 2024). A more detailed understanding of TET enzyme kinetics, locus specificity, and regulatory networks may enable the development of therapeutic strategies that actively “erase” inflammatory memory at the epigenomic level, rather than merely suppressing ongoing inflammatory signaling.

Regulatory T cells (Tregs) are pivotal in constraining adipose tissue inflammation. Butyrate-producing bacterial taxa facilitate Treg differentiation via inhibition of HDACs at the FOXP3 locus, thereby enhancing histone acetylation (H3K9ac, H3K27ac) at its promoter region and conserved non-coding sequences (Logan et al., 2016). In individuals with obesity who exhibit reduced abundance of *Faecalibacterium prausnitzii*, FOXP3 acetylation is diminished, leading to decreased Treg frequencies and exacerbation of adipose tissue inflammation. Moreover, maternal consumption of high-fiber diets during pregnancy increases fetal acetate concentrations that traverse the placental barrier, promoting differentiation of FOXP3^+^ Tregs in the offspring through elevated histone acetylation at the FOXP3 promoter. This represents a paradigm of developmental epigenetic programming with enduring consequences for metabolic health (Logan et al., 2016).

Bile salt hydrolase (BSH) activity exhibits pronounced taxon-specific variation, with direct implications for bile acid (BA)-mediated epigenetic regulation. BSH enzymes encoded by members of the Firmicutes and Actinobacteria phyla deconjugate a broad spectrum of bile salts, encompassing both glycine- and taurine-conjugated species, whereas Bacteroidetes-derived BSH activity is largely restricted to tauro-conjugated substrates (Alkhammash et al., 2022). This differential substrate specificity modulates the composition of the secondary BA pool entering the enterohepatic circulation and, consequently, the relative activation of the nuclear receptor FXR versus the G protein coupled receptor TGR5. Elevated levels of deoxycholic acid (DCA), arising from Firmicutes-dominated deconjugation, preferentially engage FXR signaling pathways and are proposed to suppress histone acetylation at chromatin regions associated with lipogenic genes. In contrast, lithocholic acid (LCA) more potently activates TGR5, thereby inducing cAMP-PKA-CREB signaling and promoting the recruitment of histone acetyltransferases (HATs) to genomic loci regulating energy expenditure (Liu, 2025). Together, these observations indicate that the taxonomic composition of BSH-expressing bacterial communities plays a determinative role in specifying which chromatin remodeling and transcriptional regulatory pathways are engaged.

Within the developmental origins of health and disease (DOHaD) paradigm, particular genomic loci have been identified as being especially vulnerable to microbiome-mediated epigenetic modulation. In rodent models, maternal consumption of a high-fat diet increases histone H3 lysine 14 (H3K14) acetylation in fetal hepatic and hypothalamic tissue, with these epigenetic alterations persisting into adulthood even following postnatal dietary normalization (Krautkramer et al., 2017). Similarly, gestational protein restriction induces intrauterine growth restriction and is associated with decreased expression of specific IGF1 mRNA isoforms, concomitant with reduced histone H3 acetylation at the IGF1 promoter in postnatal liver (Indrio et al., 2017). Gestational diabetes mellitus has been linked to differential DNA methylation in cord blood at loci such as OR2L13 and CYP2E1 (Al Bekai et al., 2025). In addition, maternal gut microbiota composition modulates methylation of the PDX1 gene crucial for pancreatic β-cell development via regulation of folate and S-adenosylmethionine (SAM) availability; excessive methyl donor supply promote PDX1 promoter hypermethylation and thereby compromise β-cell function (Chen et al., 2023). Furthermore, genetic variation in FUT2 influences colonization by folate-producing Bifidobacterium species, with non-secretor status being associated with diminished SAM-dependent methylation capacity at promoters of key metabolic genes (Iglesia Altaba et al., 2022).

Mode of delivery interacts with early postnatal nutrition to shape infant epigenomic profiles. Infants born by cesarean section display altered initial gut colonization characterized by a reduced abundance of human milk oligosaccharide (HMO)-adapted Bifidobacterium species; these microbiome differences are associated with global hypomethylation signatures in leukocyte DNA of surgically delivered neonates relative to vaginally delivered counterparts receiving comparable breastfeeding regimens (Lee, 2019; Nelson and Friedman, 2024). Breastfeeding facilitates colonization by folate-producing Bifidobacterium, which helps sustain S-adenosylmethionine (SAM)-dependent DNA methylation pathways, whereas microbial fermentation of HMOs to butyrate mediates HDAC inhibition in intestinal epithelial cells, leading to increased H3K9 acetylation at loci encoding tight junction components (e.g., claudin, occludin) and improved epithelial barrier function (Indrio et al., 2017). These early-life epigenetic alterations are hypothesized to contribute mechanistically to the epidemiologically well-established protective effects of breastfeeding against subsequent obesity and autoimmune diseases.

### 5.2 Animal models of obesity-associated dysbiosis

Animal models frequently employ exposure to a high-fat diet (HFD) to induce obesity and to investigate associated alterations in the gut microbiota and their downstream effects on host physiology (Kim et al., 2012). Lee et al. (2023) study explicitly investigates combined sleep deprivation and HFD-induced obesity, employing fecal shotgun sequencing to identify key mediators in the microbiota–gut–brain axis under these stressors. Another experimental report by Rehman et al. (2022) characterizes obesity as a chronic metabolic disorder with chronic inflammation, gut dysbiosis, and colonic hyperpermeability, and tests whether *Morchella esculenta* polysaccharide supplementation attenuates obesity parameters and reduces colonic inflammation via regulation of gut microbiota dysbiosis in an HFD-induced obesity. A further mouse study evaluates differential effects of HFD on salivary and gut microbiota while confirming obesity via body weight and blood glucose testing, extending the dysbiosis framework beyond the gut to consider oral–gut microbial differences in obesity models (Bai et al., 2025). Together, these studies align with review-level claims that dysbiosis mechanisms include barrier dysfunction and inflammation coupled to metabolic abnormalities, while also illustrating that “obesity-associated dysbiosis” may involve multiple body-site microbiomes and multiple host axes.

### 5.3 Historical and clinical evidence for microbiome–obesity associations

In their landmark study, Ley et al. (2006) examined the gut microbiota of genetically obese mice (ob/ob) and lean mice (ob/+). They found that the gut microbiota of the obese animals had 50% less Bacteroidetes and a proportionately higher number of Firmicutes. It was discovered that this feature is transmissible since colonizing germ-free mice with a “obese microbiota” increased total body fat and insulin resistance more than colonizing them with a “lean microbiota” (Turnbaugh et al., 2006). These results point to a connection between intestinal dysbiosis, a unique gut microbial profile that contributes to the development of obesity and insulin resistance, and obesity. Similar findings were corroborated by further investigations conducted on obese humans, who had higher Firmicutes and less Bacteroidetes than lean people (Ley et al., 2006). Additionally, research on obese individuals on low-calorie diets revealed a change toward a higher relative abundance of Bacteroidetes and a lower relative abundance of Firmicutes. Furthermore, Furet et al. (2010) study examined how the gut microbiota of individuals with obesity was modulated following Roux-en-Y gastric bypass (RYGB) surgery. Before RYGB, the relative abundance of the Bacteroides/Prevotella group was reduced in obese participants; however, an increase in this bacterial group was observed 3 months postoperatively. In addition, the abundance of *Escherichia coli* species increased at the three-month time point (M3) and exhibited an inverse correlation with circulating leptin concentrations and fat mass. Conversely, lactic acid bacteria declined at M3 after surgery, including members of the genus Bifidobacterium as well as the *Lactobacillus*–*Leuconostoc*–*Pediococcus* group. In an additional investigation involving obese or overweight participants undergoing an energy-restricted dietary regimen, individuals presenting with higher baseline fecal abundances of *Lactobacillus/Leuconostoc/Pediococcus* exhibited attenuated weight loss and experienced more rapid weight regain following a subsequent weight-stabilization phase (Kong et al., 2013). Moreover, a 9-year longitudinal follow-up demonstrated that obese adults with lower gut bacterial richness exhibited a greater overall increase in body weight, and an elevation in total adiposity was associated with reduced bacterial richness. These findings suggest that gut microbiota composition may play a critical role in the management of obesity and may serve as a predictor of weight-loss outcomes in response to dietary interventions (Le Chatelier et al., 2013). Finally, studies investigating alterations in the composition of the intestinal microbiota and its involvement in metabolic processes and epigenetic regulation are summarized in Table 2.

**Table 2:** Overview of gut microbiota taxa associated with obesity and related metabolic dysfunctions.

Bacterial genus/phylum	Dysbiosis direction	Related conditions	Main mechanism	Reference
Proteobacteria (phylum)	Increased	Obesity, metabolic endotoxemia	LPS production: Primary source of endotoxin driving systemic inflammation	Xu et al. (2022)
Firmicutes (phylum)	Increased (often)	Obesity, energy harvest	Energy extraction: Increased breakdown of dietary polysaccharides into energy	Turnbaugh et al. (2006)
Bacteroidetes (phylum)	Decreased (often)	Obesity (protective)	Anti-inflammatory: Often associated with lean phenotype	Ley et al. (2006)
*Faecalibacterium prausnitzii*	Decreased	T2D, obesity	Butyrate production: Anti-inflammatory; improves insulin sensitivity	Xuan et al. (2023)
*Akkermansia muciniphila*	Decreased	Obesity, insulin resistance	Mucus integrity: Protects gut barrier; prevents endotoxemia	Everard et al. (2013) and Dao et al. (2016)
*Alistipes* spp.	Decreased	Metabolic health	Metabolic protection: Consistently reduced in obesity; linked to healthy glucose	Xu et al. (2022)
*Prevotella* spp.	Varies	Obesity (west) vs. lean (east)	Diet-dependent: Pathogenic in western diet; protective in high-fiber diets	Xu et al. (2022)
*Ruminococcus* spp.	Varies	Obesity (west) vs. lean (east)	Substrate-dependent: Can be mucolytic (harmful) or starch-degrading (beneficial)	Kasai et al. (2015)
*Bifidobacterium* spp.	Decreased	Obesity, T2D	Barrier support: Cross-feeding with butyrate producers	Sasidharan Pillai et al. (2024)
*Lactobacillus* spp.	Increased	Obesity (strain-dependent)	Strain specificity: *L. reuteri* associated with obesity; others protective	Million et al. (2012)
*Fusobacterium* spp.	Increased	Inflammation	Pathogenicity: Pro-inflammatory oral bacteria found enriched in obese gut	Xu et al. (2022)
*Blautia* spp.	Increased	Visceral fat	Inflammation: Strongly correlated with visceral fat area	Xu et al. (2022)
*Oscillospira* spp.	Decreased	Leanness	Heritable: Strongly associated with leanness; difficult to culture	Xu et al. (2022)
Christensenellaceae	Decreased	Low BMI, heritability	Heritability: Most heritable taxon; promotes leanness in mice	Xu et al. (2022)
*Escherichia*/*Shigella*	Increased	Endotoxemia	LPS source: Opportunistic pathogen driving metabolic endotoxemia	Xu et al. (2022)
*Roseburia* spp.	Decreased	T2D, obesity	Butyrate production: Reduced levels linked to T2D	Qin et al. (2012)
*Eubacterium* spp.	Decreased	Metabolic health	Butyrate production: Key beneficial genus often depleted	Le Chatelier et al. (2013)

**Figure 7:**
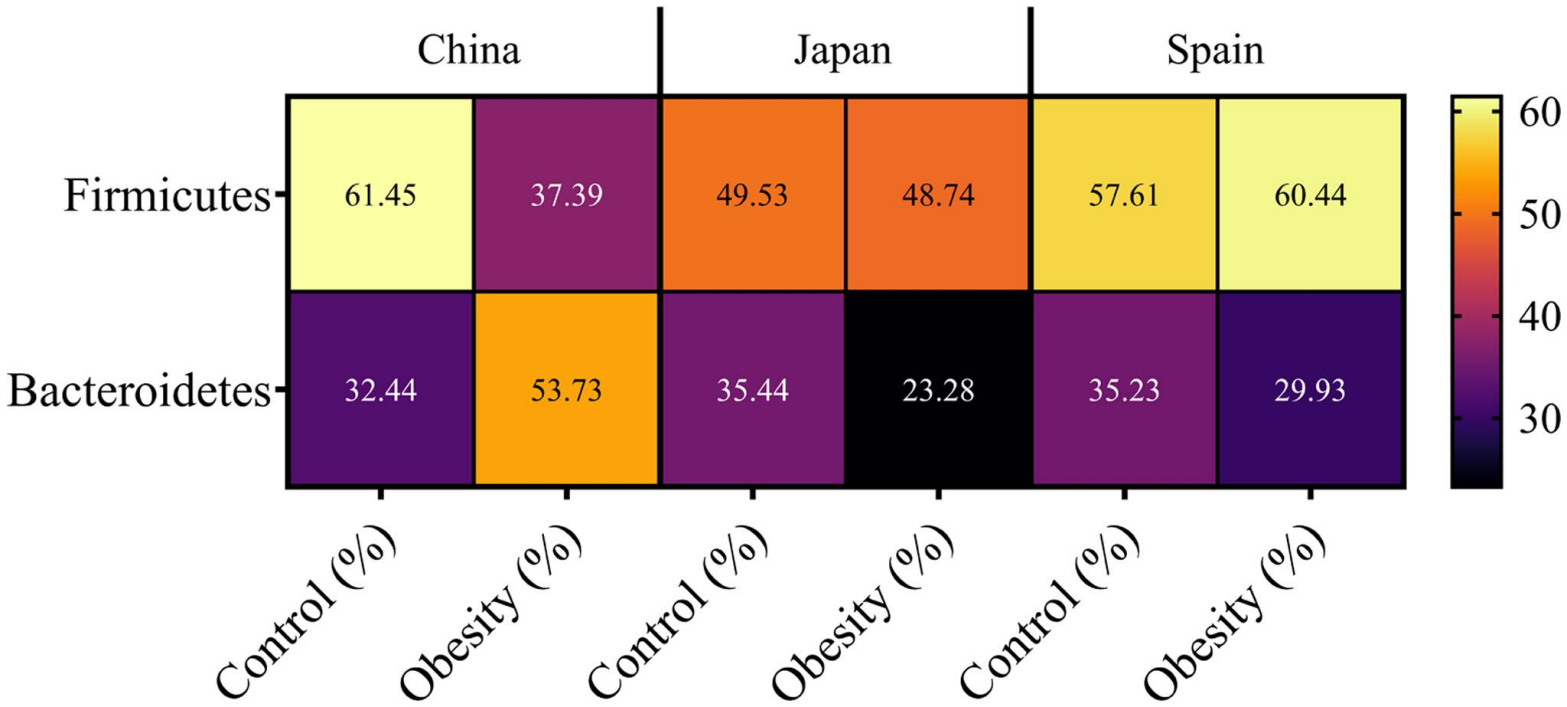
Relative abundances of Firmicutes and Bacteroidetes in control and obese individuals from China, Japan, and Spain. Cross-population differences should be interpreted cautiously because obesity-associated patterns are heterogeneous.

Through systematic screening of the previous studies, some of the relationship between the gut microbiome and obesity was identified and summarized in Figure 7. In the Chinese cohort, the relative abundance of Firmicutes was substantially lower in individuals with obesity (37.39%) compared with normal-weight controls (61.45%), whereas Bacteroidetes exhibited the opposite pattern, with a higher relative abundance in obese subjects (53.73%) than in controls (32.44%). The combined relative abundance of these two dominant phyla was comparable between groups (93.89% in controls vs. 91.12% in obese individuals), indicating that Firmicutes and Bacteroidetes together continued to constitute the majority of the gut microbiota in both phenotypes (Duan et al., 2021). Among Japanese participants, the relative abundance of Firmicutes was similar between groups (49.53% in controls vs. 48.74% in individuals with obesity), whereas the relative abundance of Bacteroidetes was markedly lower in the obese group (23.28%) compared with controls (35.44%). As a result, the combined proportion of Firmicutes and Bacteroidetes decreased from 84.97% in the control group to 72.02% in the obese group, indicating a relatively increased contribution of non-dominant phyla to the overall gut microbiota in obese Japanese subjects (Kasai et al., 2015). In the Spanish cohort, individuals with obesity demonstrated a marginally higher relative abundance of Firmicutes (60.44%) compared with normal-weight controls (57.61%), together with a modest reduction in Bacteroidetes (29.93% vs. 35.23% in controls). The combined relative abundance of these two phyla was high in both groups and only slightly reduced in the obese group (92.84% in controls vs. 90.37% in individuals with obesity) (Companys et al., 2021). Taken together, these findings indicate that obesity is associated with alterations in the relative abundances of Firmicutes and Bacteroidetes; however, the direction and magnitude of these shifts are population dependent. In the Chinese cohort, obesity is characterized by a decreased relative abundance of Firmicutes and an enrichment of Bacteroidetes, resulting in a lower Firmicutes/Bacteroidetes ratio in individuals with obesity. By contrast, the Japanese and Spanish cohorts exhibit the more commonly reported pattern, in which obesity is associated with a relative depletion of Bacteroidetes and a stable or modestly increased proportion of Firmicutes, producing a higher Firmicutes/Bacteroidetes ratio in obese groups. The cumulative relative abundances demonstrate that, despite these compositional changes, Firmicutes and Bacteroidetes remain the predominant bacterial phyla across all countries studied. Nevertheless, the more pronounced reduction in the cumulative proportion of these two phyla in obese Japanese subjects suggests an expansion of additional phyla (e.g., Proteobacteria, Actinobacteria) in this group, which contribute to obesity-associated dysbiosis. Although the Firmicutes/Bacteroidetes ratio has historically been discussed as a potential feature of obesity-associated dysbiosis, this metric remains controversial and is not consistently reproduced across studies or populations. As illustrated by the contrasting findings across Chinese, Japanese, and Spanish cohorts in the present review, the direction and magnitude of phylum-level shifts are context dependent and likely influenced by diet, geography, host characteristics, and analytical methodology. Moreover, interpretation at the phylum level may obscure substantial functional heterogeneity within both Firmicutes and Bacteroidetes, as taxa within the same phylum can differ markedly in metabolic activity, mucosal interactions, and inflammatory relevance. Therefore, phylum-level ratios should be interpreted cautiously and complemented by genus-, species-, and function-level analyses.

On the other hand, Table 3 summarizes the gut bacterial genera detected in adults with type 2 diabetes mellitus (T2DM) and in non-diabetic controls, grouped by phylum as screened by Umirah et al. (2021). At the Firmicutes phylum level, both groups harbor a broad diversity of genera, but the composition differs. In T2DM, detected Firmicutes genera include *Abiotrophia*, *Anaerotruncus*, *Bacillus*, *Bulleidia*, *Dorea*, *Holdemania*, *Lactobacillus*, *Oscillibacter*, *Peptostreptococcus*, *Ruminococcus*, *Sporobacter*, and *Subdoligranulum*. In controls, the Firmicutes profile is instead characterized by *Anaerostipes*, *Blautia*, *Coprococcus*, *Faecalibacterium*, *Lachnospira*, *Megamonas*, *Megasphaera*, *Roseburia*, and *Veillonella*. Within Bacteroidetes, T2DM samples are dominated by *Prevotella* and *Parabacteroides*, whereas control subjects show *Bacteroides* and *Alistipes*. For Actinobacteria, T2DM is associated with the genera *Bifidobacterium*, *Collinsella*, and *Eggerthella*, while in controls only *Atopobium* is listed. The Proteobacteria phylum in T2DM includes *Escherichia* (and broader *Enterobacteriaceae*), *Bilophila*, *Desulfovibrio*, and *Pseudomonas*; in contrast, only *Haemophilus* is reported among Proteobacteria in controls. Fusobacteria (*Fusobacterium*) appear only in T2DM. Finally, Verrucomicrobia is represented by *Akkermansia* in both T2DM and control groups. At the Firmicutes level, the diabetic microbiota appears qualitatively shifted from a community dominated by classical butyrate-producing, anti-inflammatory genera toward a more heterogeneous set including several genera previously linked to dysbiosis (James et al., 2022). In the control group, many Firmicutes genera (*Faecalibacterium*, *Roseburia*, *Anaerostipes*, *Coprococcus*, *Megasphaera*, *Lachnospira*) are well-known short-chain fatty acid (especially butyrate) producers that support epithelial integrity and exert anti-inflammatory effects (Fusco et al., 2023). In the T2DM group, these taxa are replaced or overshadowed by genera such as *Dorea*, *Ruminococcus*, *Peptostreptococcus*, and *Lactobacillus*, several of which have been associated in other studies with pro-inflammatory profiles or altered energy harvest in metabolic disease (Donati Zeppa et al., 2024; Sinisterra Loaiza et al., 2025). Thus, even within the same phylum, the functional potential of the Firmicutes community appears less favorable in T2DM than in controls. Within Bacteroidetes, controls show a signature dominated by *Bacteroides* and *Alistipes*, genera that have frequently been associated with metabolic health and efficient fermentation of complex polysaccharides (Shin et al., 2024). In contrast, T2DM samples feature *Prevotella* and *Parabacteroides*, depending on diet and strain, *Prevotella* can either be beneficial (fiber-rich, plant-based diets) or associated with inflammatory states; its prominence here, together with *Parabacteroides*, suggests a reconfiguration of Bacteroidetes away from the classic *Bacteroides*-dominated pattern of many healthy Western microbiomes (Precup and Vodnar, 2019; Tsai et al., 2023). This shift may reflect differences in substrate use and in the profile of produced metabolites in T2DM. The Actinobacteria findings indicate a broader and potentially more dysbiotic actinobacterial community in T2DM, whereas only *Atopobium* is reported in controls, T2DM samples contain *Bifidobacterium*, *Collinsella*, and *Eggerthella*. *Bifidobacterium* is often considered beneficial, but its presence here alongside *Collinsella* and *Eggerthella* genera repeatedly linked to cardiometabolic risk and low-grade inflammation (Valassi et al., 2023), suggests that the Actinobacteria phylum in T2DM contain a mixture of protective and adverse members, with a net tilt toward a more pro-inflammatory configuration (Garcia-Gutierrez et al., 2024).

**Table 3:** Comparison of gut bacterial genera by phylum in individuals with type 2 diabetes mellitus versus non-diabetic controls.

Phylum	T2DM	Control
Firmicutes	*Abiotrophia*, *Anaerotruncus*, *Bacillus*, *Bulleidia*, *Dorea*, *Holdemania*, *Lactobacillus*, *Oscillibacter*, *Peptostreptococcus*, *Ruminococcus*, *Sporobacter*, *Subdoligranulum*	*Anaerostipes*, *Blautia*, *Coprococcus*, *Faecalibacterium*, *Lachnospira*, *Megamonas*, *Megasphaera*, *Roseburia*, *Veillonella*
Bacteroidetes	*Prevotella*, *Parabacteroides*	*Bacteroides*, *Alistipes*
Actinobacteria	*Bifidobacterium*, *Collinsella*, *Eggerthella*	*Atopobium*
Proteobacteria	*Escherichia*, *Enterobacteriaceae*, *Bilophila*, *Desulfovibrio*, *Pseudomonas*	*Haemophilus*
Fusobacteria	*Fusobacterium*	—
Verrucomicrobia	*Akkermansia*	*Akkermansia*

### 5.4 Safety considerations for microbiome-directed epigenetic interventions

The therapeutic exploitation of microbiome–epigenetic interfaces must be carefully balanced against substantial safety concerns deriving from the pleiotropic functions of epigenetic enzymes beyond metabolic regulation. Global histone deacetylase inhibition resulting from sustained exposure to elevated butyrate concentrations has the potential to deregulate proto-oncogene expression or to silence tumor suppressor genes, particularly under conditions of excessive methyl-donor availability (Rukavina Mikusic et al., 2025). The HDAC inhibitory activity of butyrate is both concentration-dependent and broadly non-selective; prolonged colonic exposure may therefore facilitate oncogenic transformation in predisposed individuals, especially those harboring pre-existing mutations in Wnt signaling components or DNA repair pathways. Similarly, non-targeted elevation of secondary bile acids, such as that induced by bile salt hydrolase-active probiotic supplementation or diet-induced shifts in bile acid metabolism precipitate cholestatic injury or promote tumorigenesis, contingent on the prevailing epigenetic landscape (Liu, 2025). Deoxycholic acid, in particular, has been associated with DNA damage and the promotion of hepatocellular carcinoma when present at supraphysiological concentrations. Excessive methyl-donor supply arising from high-dose folate supplementation in combination with folate-producing probiotics may drive inappropriate hypermethylation of loci essential for pancreatic β-cell function (e.g., PDX1), tumor suppression (e.g., CDKN2A/p16, MLH1), or lipid metabolism (e.g., LEP), thereby exacerbating metabolic dysregulation or increasing oncogenic risk (Lizarraga et al., 2024). Since the one-carbon metabolism pathway integrates diverse dietary inputs into a tightly regulated network, interventions directed at isolated components without regard for systemic cofactor balance may produce unintended, locus-specific epigenetic alterations. Broad-spectrum TLR4 inhibition-proposed as a strategy to prevent LPS-induced inflammatory epigenetic remodeling carries the risk of attenuating essential antimicrobial immune responses, thereby heightening susceptibility to infection (Liu, 2025). Likewise, excessive anti-inflammatory bias induced by HDAC activation or histone acetyltransferase inhibition has the potential to compromise host readiness to respond to pathogenic challenges. Engineered probiotic approaches must rigorously account for colonization dynamics, ecological competition within resident microbial communities, and the potential for horizontal gene transfer prior to clinical deployment. Persistent colonization by the intended strains is required to sustain altered metabolite profiles sufficiently long to establish stable epigenetic reprogramming; however, many hosts exhibit reversion toward baseline microbiome configurations within months in the absence of continued provision of appropriate dietary substrates.

## 6 Conclusion

In summary, current evidence supports a biologically plausible and increasingly well-characterized interplay between the gut microbiome and host epigenetic regulation in obesity and type 2 diabetes mellitus, with microbial metabolites such as short-chain fatty acids, folate-related one-carbon intermediates, bile acid derivatives, and other signaling molecules influencing DNA methylation, histone modifications, and non-coding RNA pathways linked to inflammation, insulin sensitivity, and energy homeostasis. At the same time, this literature remains marked by important limitations, including reliance on observational designs, inconsistent taxonomic signatures across cohorts, methodological variability in microbiome profiling, and incomplete integration of microbial composition with functional metabolite output and host epigenetic readouts. Taken together, the available data suggest that the gut microbiome–epigenetic axis is not a simple linear pathway but a context-dependent and bidirectional network shaped by diet, host genetics, developmental exposures, medication history, and baseline microbial ecology. Although mechanistic studies in animal models and controlled experimental systems support causal potential, human translation remains incomplete, and many reported associations in obesity and diabetes should still be interpreted cautiously until validated in longitudinal and interventional settings. Several research priorities should now be considered central for advancing this field. First, future studies must determine whether microbiome alterations in obesity and type 2 diabetes are causal drivers, downstream consequences, or context-dependent amplifiers of host epigenetic dysregulation. Second, research should identify which microbiota-derived metabolites and metabolite combinations reproducibly modulate specific epigenetic enzymes, including DNMTs, HDACs, HATs, and TET enzymes, in metabolically relevant tissues. Third, longitudinal studies are needed to establish whether microbiome-associated epigenetic modifications are transient, reversible, or stably maintained over time, particularly after dietary, probiotic, postbiotic, or fecal microbiota-based interventions. Fourth, population-specific mapping is essential to clarify how diet, host genetics, antibiotic exposure, medication use, and baseline microbiome structure contribute to heterogeneity in metabolic and epigenetic responses. Methodological innovation will be equally important. Progress will depend on harmonized multi-center study designs that integrate strain-resolved metagenomics, targeted metabolomics, proteomics of epigenetic enzyme activity, and locus-specific epigenomic profiling rather than relying on taxonomic composition alone. Future intervention studies should incorporate direct functional validation through matched metabolite quantification, chromatin mark mapping, standardized metabolic phenotyping, and repeated sampling before and after intervention to improve causal inference. In parallel, translational research should prioritize safety-oriented frameworks, including surveillance for off-target chromatin remodeling and other unintended genomic consequences associated with broad microbiome-based epigenetic manipulation. Overall, the field is moving toward a more mechanistic and clinically relevant understanding of how microbial ecology interfaces with host gene regulation in metabolic disease. With standardized methodology, deeper functional resolution, and more rigorous causal designs across diverse populations, the microbiome–epigenetic axis may become a meaningful foundation for precision prevention and therapy in obesity and type 2 diabetes, but its clinical application still requires careful validation before broad implementation.

## Glossary

5hmC: 5-Hydroxymethylcytosine
5mC: 5-Methylcytosine
BA: Bile acid
BSH: Bile salt hydrolase
CREB: cAMP response element-binding protein
DCA: Deoxycholic acid
DNA: Deoxyribonucleic acid
DNMT: DNA methyltransferase
DNMT1: DNA methyltransferase 1
DNMT3A: DNA methyltransferase 3A
FGF21: Fibroblast growth factor 21
FOXP3: Forkhead box P3
FXR: Farnesoid X receptor
GLP-1: Glucagon-like peptide-1
GLUT4: Glucose transporter type 4
GPR41: G-protein-coupled receptor 41
H3K27ac: Histone H3 lysine 27 acetylation
HAT: Histone acetyltransferase
HCA: Hyocholic acid
HDAC: Histone deacetylase
HDAC3: Histone deacetylase 3
HDAC6: Histone deacetylase 6
HFD: High-fat diet
HMO: Human milk oligosaccharide
LCA: Lithocholic acid
LPS: Lipopolysaccharide
miRNA: MicroRNA
NLRP3: NLR family pyrin domain containing 3
RNA: Ribonucleic acid
RYGB: Roux-en-Y gastric bypass
SAM: S-adenosyl-L-methionine
SCFA: Short-chain fatty acid
T2D: Type 2 diabetes
T2DM: Type 2 diabetes mellitus
TET: Ten-eleven translocation
TGR5: Takeda G-protein-coupled receptor 5
TLR4: Toll-like receptor 4
