# Supplementary material for: Gut microbiome–epigenetic crosstalk in obesity and type 2 diabetes: mechanisms, evidence, and translational opportunities
Source: Front Microbiol. 2026 Mar 31;17:1805937. doi: 10.3389/fmicb.2026.1805937 (PMC13076291; doi:10.3389/fmicb.2026.1805937)
Supplement: Supplementary file 1 [file Data_Sheet_1.pdf]

Gut Microbiome–Epigenetic Crosstalk in Obesity and Type 2 Diabetes: Mechanisms, Evidence, and Translational Opportunities

Seham Saeed Alzahrani [1,*]

[1.] Department of Biotechnology, College of Science, Taif University, P.O. Box 11099, Taif 21944, Saudi Arabia.

Bibliometric Overview

Furthermore, as illustrated in (Figure S1), the annual number of publications addressing the gut microbiome, epigenetic mechanisms, and human disease exhibits a continuous and marked increase between 2016 and 2025. In 2016, only 33 articles were published, and the output remained relatively low through 2018, with 46 and 43 publications in 2017 and 2018, respectively, indicative of an initial exploratory phase of this research domain. From 2019 onward, publication volume began to accelerate, increasing to 71 papers in 2019 and 78 in 2020, followed by a pronounced rise to 128 articles in 2021, which appears to represent a transition from a slow-growth to a rapid-expansion phase. This upward trajectory persisted in 2022 and 2023, with 134 and 140 publications, respectively, and intensified further in 2024, when 180 articles were published, before reaching a maximum of 300 publications in 2025. In total, the temporal distribution of publications approximates an exponential growth pattern, consistent with the high annual growth rate calculated for this dataset. This pattern suggests that the interface between gut microbiome research and epigenetics in human disease has evolved from a niche area into a major focus of scientific inquiry over the past decade. The comparatively low output prior to 2019 implies that the conceptual and mechanistic connections between microbial communities and epigenetic regulation were still being formulated and evaluated within specific disease settings. The sustained increase from 2019 onward likely reflects accumulating evidence for causal and mechanistic interactions, which has catalyzed broader engagement across oncology, metabolic disease, neurology, and immune-mediated disorders. The particularly steep rise from 2021 to 2025 indicates that this field has become highly interdisciplinary, drawing researchers from microbiology, epigenetics, systems biology, and diverse clinical specialties, and suggests that, if current trends persist, annual publication output will continue to expand in the coming years. (Tian and Chen 2024).

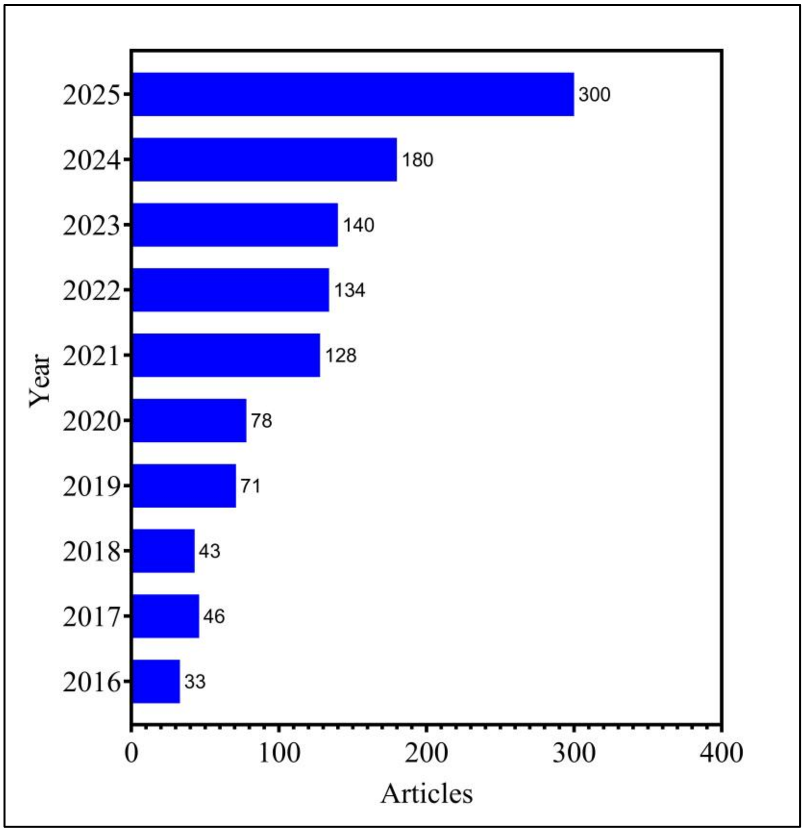

Figure S1. Annual number of Scopus-indexed publications on gut microbiome/gut microbiota and epigenetic mechanisms in human diseases from 2016 to 2025.

While, (Figure S2) presents a three-field plot that depicts the interrelationships among countries, prolific authors, and author keywords within the literature on gut microbiome–epigenetic mechanisms in human disease. On the left side of the plot, China emerges as the predominant contributing country, followed by the United States, and, to a substantially lesser degree, the United Kingdom, Australia, Japan, Korea, the Netherlands, and France. This distribution indicates that research output in this domain is highly concentrated in a limited set of nations, with China and the United States functioning as the principal production hubs. In the central panel, the most prolific authors (e.g., Li Y, Wang X, Zhang Y, Wang Y, Li X, Wang Z, Chen Y) are primarily affiliated with Chinese institutions. This pattern underscores China's central role in this research area and is consistent with trends documented in related bibliometric analyses of gut microbiome and epigenetics. The right panel demonstrates that these authors most frequently employ keywords such as "gut microbiota/gut microbiome," "inflammation," "epigenetics," "obesity," "short-chain fatty acids," "colorectal cancer," "DNA methylation," "gut-

brain axis," "probiotics," and "metabolites," thereby delineating the principal thematic foci of the field. Collectively, this configuration suggests that the intellectual structure of research on the gut microbiome and epigenetics is organized around a Sino-centric author network with a strong emphasis on metabolic, inflammatory, oncologic, and neuro-immune pathways. The pronounced prominence of keywords related to short-chain fatty acids, DNA methylation, and obesity indicates that mechanistic investigations into microbial metabolites and host epigenetic regulation of metabolism and cancer risk constitute a major research frontier. The discernible, albeit comparatively smaller, contributions from the United States and various European and Asia–Pacific countries, coupled with the shared use of core terms such as "gut microbiota," "epigenetics," and "inflammation," point to an emerging yet still asymmetrical landscape of international collaboration. Within this landscape, a relatively small cohort of highly productive Chinese authors generates a substantial proportion of the scholarly output while maintaining connections to a broader, globally distributed community of researchers (Yue et al., 2020).

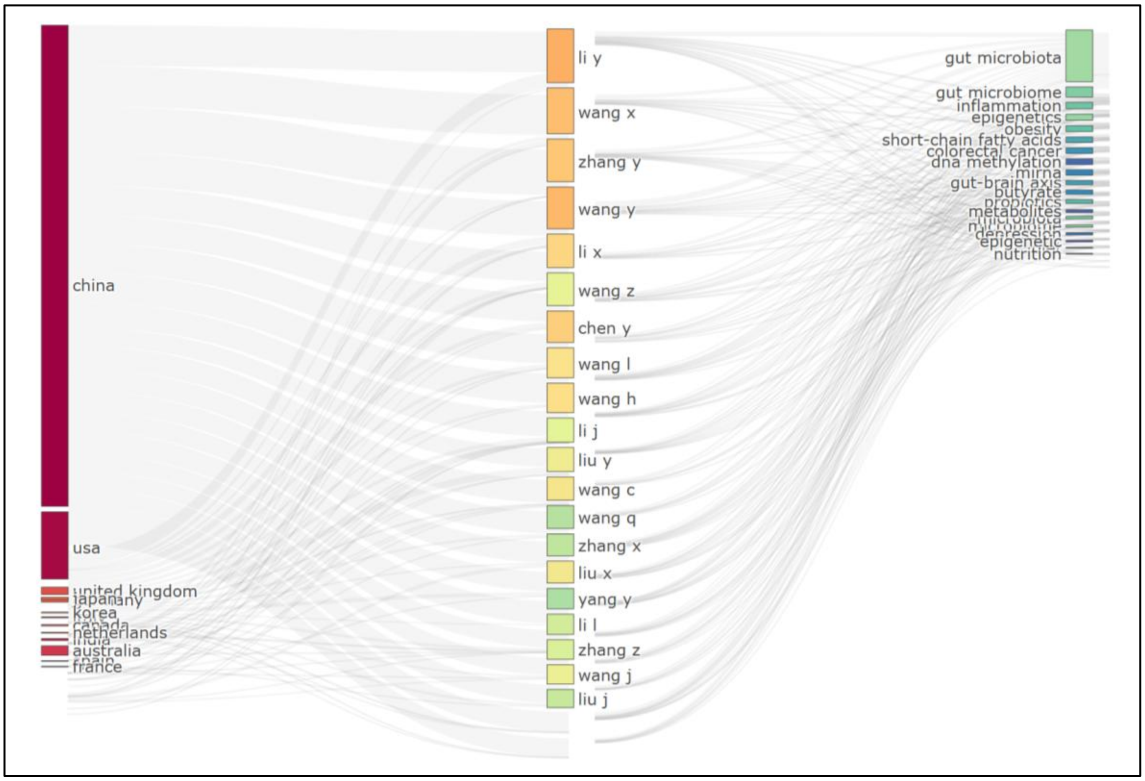

Figure S2. Three-field plot linking authors' countries (left), most productive authors (middle), and author keywords (right) for publications on gut microbiome and epigenetic mechanisms in human diseases.

Moreover, as illustrated in (Figure S3), the United States and China emerge as the largest and most centrally positioned nodes, reflecting both the highest levels of research output and extensive collaborative ties with a wide range of partner countries across the network. European countries particularly the United Kingdom, Germany, Italy, France, Spain, and the Netherlands, constitute a dense, highly interconnected collaboration core, indicative of frequent multinational co-authorship within Europe and robust linkages to the United States. Additional regional clusters are also discernible, including an Asia-oriented collaboration group (e.g., China in association with South Korea, Japan, and other geographically proximate countries) and a South America-related cluster (e.g., Brazil with Argentina, and Austria appearing at the periphery), collectively illustrating collaboration patterns that are strongly shaped by geographic proximity. The configuration reflects a classic "hub-and-spoke" arrangement, characteristic of global scientific collaboration networks, in which a handful of high-output countries most notably the USA and China serve as central intermediaries linking numerous regional clusters (Isfandyari-Moghaddam et al., 2023). The compact European cluster implies strong intra-regional integration (likely facilitated by shared funding frameworks and proximity), while more peripheral nodes with fewer links reflect limited international integration or smaller research capacity in this specialized topic area.

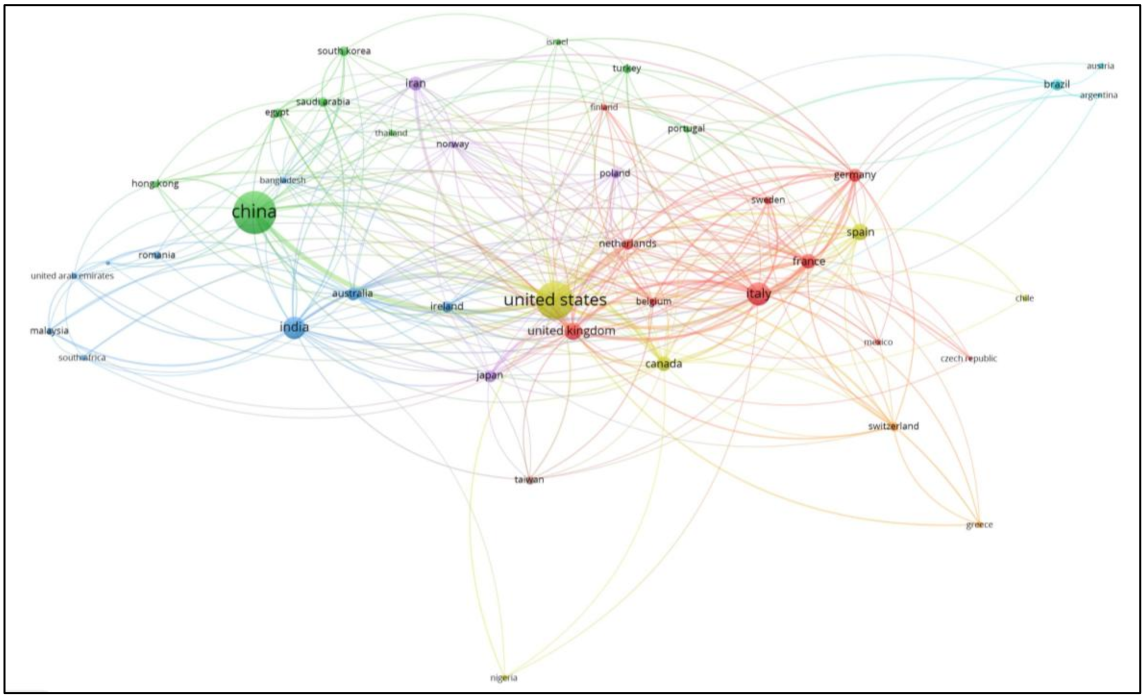

Figure S3. Country co-authorship network visualization (using VOSviewer) for publications on the gut microbiome/gut microbiota and epigenetic mechanisms in human diseases (2016-2025), including countries with ≥5 documents; node size indicates productivity, and links indicate international co-authorship strength.
